# Three-Dimensional Printing for Cancer Applications: Research Landscape and Technologies

**DOI:** 10.3390/ph14080787

**Published:** 2021-08-10

**Authors:** Ruixiu Li, Yu-Huan Ting, Souha H. Youssef, Yunmei Song, Sanjay Garg

**Affiliations:** Pharmaceutical Innovation and Development (PIDG) Group, Clinical and Health Sciences, University of South Australia, Adelaide, SA 5000, Australia; ruixiu.li@mymail.unisa.edu.au (R.L.); tinyy011@mymail.unisa.edu.au (Y.-H.T.); souha.youssef@mymail.unisa.edu.au (S.H.Y.); May.Song@unisa.edu.au (Y.S.)

**Keywords:** 3D printing, cancer, personalisation, dosage form, 3D bioprinting, medical device

## Abstract

As a variety of novel technologies, 3D printing has been considerably applied in the field of health care, including cancer treatment. With its fast prototyping nature, 3D printing could transform basic oncology discoveries to clinical use quickly, speed up and even revolutionise the whole drug discovery and development process. This literature review provides insight into the up-to-date applications of 3D printing on cancer research and treatment, from fundamental research and drug discovery to drug development and clinical applications. These include 3D printing of anticancer pharmaceutics, 3D-bioprinted cancer cell models and customised nonbiological medical devices. Finally, the challenges of 3D printing for cancer applications are elaborated, and the future of 3D-printed medical applications is envisioned.

## 1. Introduction

Cancer remains one of the major public health issues worldwide, with 18.1 million new cases and 9.6 million deaths globally in 2018, and an increase of 70% was predicted in the next 2 decades [1]. The literature revealed that the average efficacy rate of a cancer drug was as low as 25%, suggesting that 75% of cancer patients suffered from overdoses and potential adverse reactions [2,3]. The limited success of cancer therapy is attributed to multidrug resistance, decreased permeability of the drug, extracellular enzymatic degradation, deficiency of enzymes required to activate prodrugs and dose-limiting toxicity [4,5,6]. Furthermore, advances in basic research have created opportunities for the improvement of medicine. Hundreds of gene variations have been discovered related to human illness, and this great genetic variability is what the varied treatment responses among individual patients can be attributed to. Molecular diagnostic technologies such as microarrays, protein expression profiles and oncogenic signalling pathways have led the way towards the discovery of treatment targets and hence personalised cancer therapy [7]. Nonetheless, these tools face challenges such as cost, complex procedures, unique genomic profiles for each patient and ethical issues [8,9].

Three-dimensional printing (3DP) has been considered an industrial revolution [10] due to the ability to deliver tailored products that serve many advantages on more than one level. First, it has been established that the “one size fits all” approach is not effective when it comes to therapy owing to the variability between patients considering factors such as age, genetics, anatomy, underlying medical conditions, allergies, etc. [11,12,13]. Second, easily creating prototypes is possible for a thorough evaluation before mass production, which is also performed on the basis of demand, thus reducing waste and avoiding unnecessary over-production [14]. Additionally, 3DP offers superior solutions for the prosthetic industry owing to the ability to simulate patient-specific complex structures with high accuracy and relative ease.

This review gives a broad overview of the recent progress of 3DP in the applications of cancer research and treatment, which can be organised into several broad categories, including 3D-printed anticancer dosage forms, 3D-bioprinted cell models and customised medical devices.

## 2. Papers and Patents Related to Cancer Research Using 3D-Printing Technologies

**Figure 1:**
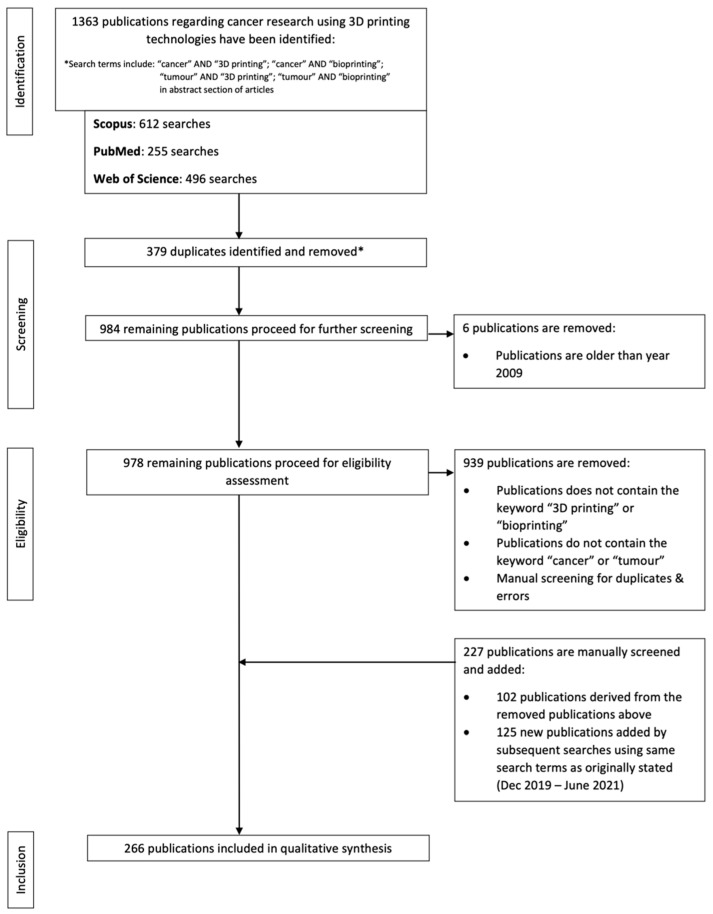
Process of identification, review and inclusion of papers published regarding cancer research using 3DP technologies (* based on EndNote X9 software).

A search for cancer research using 3DP has been conducted through three trusted sources, which include Scopus, PubMed and Web of Science. The searches are performed by identifying the terms “cancer” AND “3D printing”, “cancer” AND “bioprinting”, “tumour” AND “3D printing”, “tumour” AND “bioprinting” in the abstract of the articles in PubMed and Web of Science Core Collection using the software, EndNote™. The same search terms are performed in Scopus, and the resultant articles are imported into EndNote™. Subsequently, the searches are further narrowed down with the addition of the search terms mentioned above in the keywords section of all articles. Duplicates and publications older than the year 2009 are excluded from this study. Subsequently, 227 publications are added manually, both from the excluded data and new searches. These include a further search of the terms including “tumour”, “oncology”, “additive manufacturing” and “bioprinting”. In addition, new publications up to July 2021 have also been screened and added to the current search database. Hence, there is a final total of 266 publications included in this study (Table S1). The data are correct as of 4 July 2021. The flowchart (Figure 1) is constructed according to the PRISMA statement [15].

**Figure 2:**
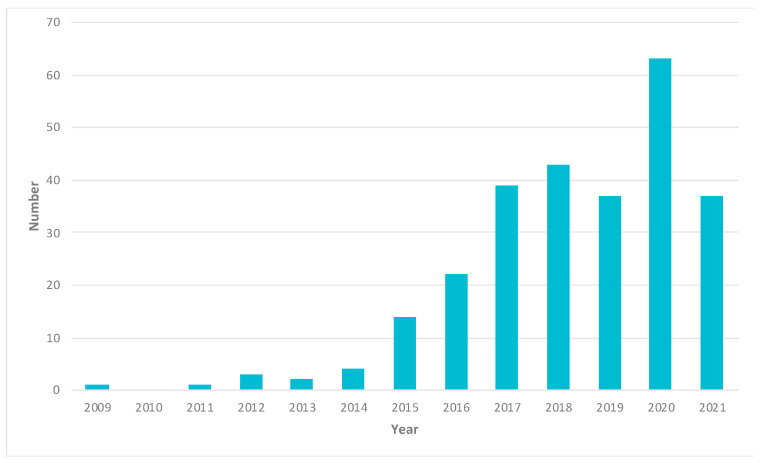
3DP in the applications of cancer according to the year of publication.

Analysis of the 266 publications (Table 1, Figure 2) regarding cancer research using 3DP technologies shows that 25.4% of the total publications are not cancer-specific or do not mention specific cancer. This is followed by breast cancer, accounting for 14.7% of the total publications. Three-dimensional printing technologies developed for brain cancer account for 8.1%. The other notable researches can be seen with bone cancer (7.7%), head and neck cancer (5.9%), gynaecological cancer (5.5%) and kidney cancer (4.8%). Among the 266 publications, only 5 publications mentioned 2 or more types of cancer.

**Figure 3:**
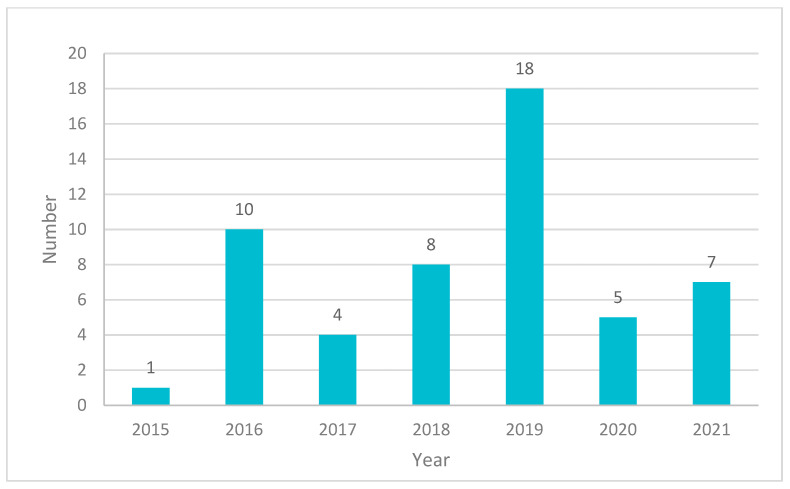
Patents related to 3DP in the applications of cancer according to the year of publication.

A search for patents relating to cancer applications using 3DP technologies has been conducted on World Intellectual Property Organisation (WIPO). The searches are performed by identifying the terms “cancer” and “3D printing”. There is a total of 51 patents included in this study (Figure 3, Table S2). The data are correct as of July 2021.

## 3. Clinical and Market Use of 3D-Printed Products for Cancer Treatment

Based on our research, there is currently no FDA approved 3D-printed drug for the treatment of cancer. Although there are various 3D-printed medical devices approved by the FDA Centre for Devices and Radiology Health (CDRH) [16], none are directly intended for cancer treatments.

However, there are implants that are approved by FDA that have the potential to repair damage caused by cancer. For instance, the SpineFab^®^ Vertebral Body Replacement (VBR) System developed by Oxford Performance Materials, Inc. obtained FDA approval through the 510(k) pathway in July 2015 [17]. The aforementioned SpineFab^®^ device is designed with the company’s custom polyetherketoneketone (PEKK) technology known as OXPEKK^®^ in tandem with proprietary 3DP technology [17]. The spinal column that is affected due to the presence of a tumour can be replaced with SpineFab^®^ device [18].

3D-printed polymeric devices for bone repairs such as OsteoFab^®^ Patient-Specific Cranial Device and OsteoFab^®^ Patient-Specific Facial Device are also approved by FDA in February 2013 and July 2014, respectively [17]. Despite that both devices are not stated to be used for cancer, these two products have great potential to repair damage caused by bone cancer due to their customizability and biocompatibility.

Three-dimensional printing of the tumour models enables the personalisation of cancer treatment [19]. The FDA outlines the regulatory requirement for 3DP of patient-specific structural models as it is classified as a Class II medical device [20]. Materialise NV is the first company to obtain FDA approval for its 3DP software, Materialise Mimics inPrint [20]. The software is designed to generate files for 3DP of structural models which can be used for surgical preparation [20].

**Figure 4:**
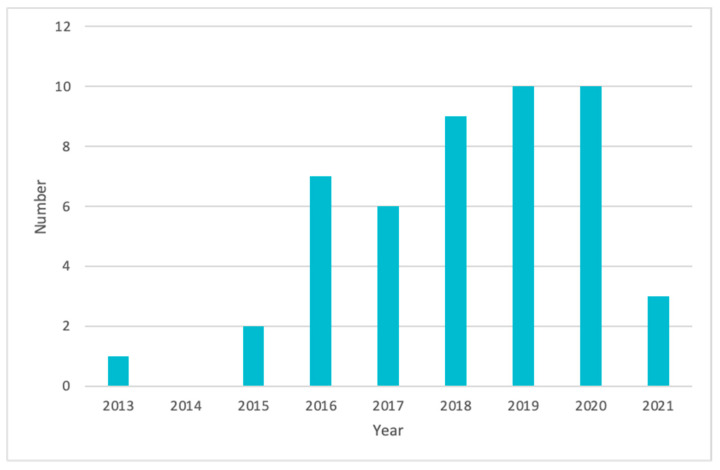
Clinical trials related to cancer utilising 3DP technologies by the year.

**Figure 5:**
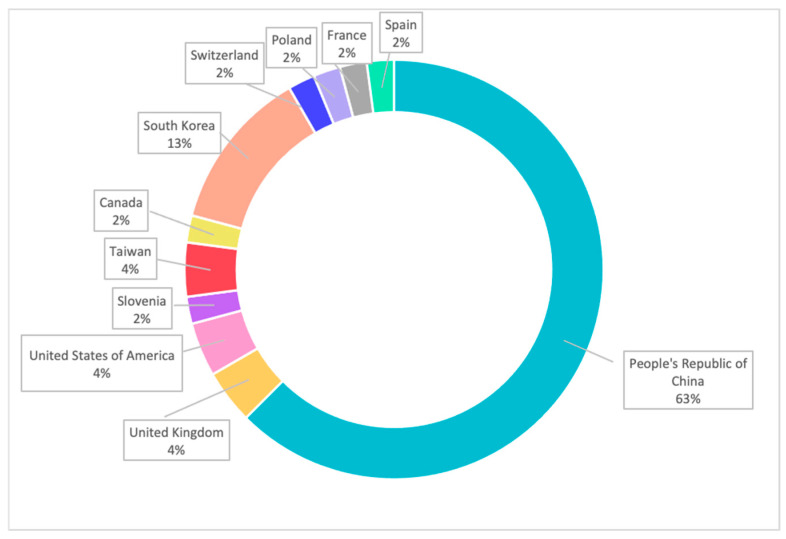
Clinical trials related to cancer utilising 3DP technologies by country.

There are 48 clinical trials in the field of oncology that utilise 3DP technologies (Figure 4, Table S3). The data are collected from the World Health Organisation Clinical Trial Registry Platform and ClinicalTrial.gov (Bethesda, MD, USA). The data are selected from the year 2013 onwards. The data are analysed using search terms including “Cancer and 3D printing”, “Neoplasm and 3D printing” and “Tumour and 3D printing”. The searches are performed and corrected as of 28 June 2021. According to the data (Figure 5), the majority of the clinical trials are conducted in the People’s Republic of China, which accounts for 63% of the clinical trials in the field of oncology. This is followed by South Korea (13%) and the United States of America, the United Kingdom and Taiwan with 4% of the total 48 clinical trials, respectively.

## 4. 3D Printing of Anticancer Dosage Forms

Since the report of the first 3D-printed pill for drug delivery purposes in 1996 [21], 3DP technologies have been increasingly utilised for pharmaceutical manufacturing, landmarked by the emergence of the first FDA approved 3D-printed medicine, SPRITAM^®^, in August 2015. A precedence has been set for the manufacture of novel dosage forms using 3DP technologies [22]. Yet so far, there is no FDA-approved 3D-printed medicine for cancer treatment in the market; many researches have been pioneered to investigate 3D-printed anticancer dosage forms (Table 2), from the local delivery implant and oral dosage form to transdermal dosage form as discussed below.

## 5. 3D Printing of Implantable Drug Delivery Devices

This cutting-edge technology has gained significant popularity and applicability in the field by imparting therapeutic effects besides providing physical support that varies from stents, scaffolds, implantable tablets, meshes and patches [23]. Three-dimensional printing has offered novel strategies for manufacturing such devices where personalisation is required as different anatomies, ages, genders and pathological conditions must be considered [24]. Currently, drug-eluting implants available on the market lack these considerations of the distinction among individual patients; however, with computer-aided design and different options of printing materials, 3DP enables the production of implants tailored to satisfy individual needs by developing products with different compositions, anatomical shapes, physical and mechanical properties and controlled drug release [2,25].

Among these implants, biodegradable polymers are extensively used as the printing matrices, such as PCL, PLA and PLGA, which are FDA-approved and well-known for their good biodegradability, biocompatibility and non-toxic properties [26]. 

### 5.1. 3D Printing of Local Chemotherapy or Thermotherapy Implants

With computer-aided design and different options of printing materials, 3DP allows the creation of personalised chemotherapeutic implants for a variety of cancers, as shown in Table 1 [2]. Three-dimension-printed implants have been widely applied in the field of bone fracture, especially fracture caused by original bone cancer or secondary cancer metastasised from other body sites [2]. In the human body, each bone has its unique anatomy characterised by varied size, shape and mechanical strength, which vary with age and gender. Thus, the treatment for bone defects and fractures caused by cancer requires adequate personalisation for each patient. Chen et al. formulated a 3D-printed tissue engineering scaffold, which not only offered mechanical support for the repair of bone defects caused by bone tumours but also performed local sustained drug release to eliminate residual cancer cells [27].

Among these implants for bone fracture repair, what is worth noting is the use of stimulus to achieve the photothermal effect, magnetic heating or magnetic modulation of release rates of 3D-printed materials [28,29,30,31,32,33]. Ma, H et al. reported the fabrication of a 3D-printed scaffold that utilised the photothermal effect of polydopamine to kill tumour cells and support attachment and proliferation of bone stem cells benefited from its mussel-inspired nanostructure surface [29]. Zhang et al. used 3DP to fabricate bifunctional scaffolds composed of magnetic iron oxide, Mechanically Bonded Graphite (MBG), Polycaprolactone and doxorubicin, which provided enhanced mechanical properties and significantly inhibited bone cancer recurrence by combining slow drug release and magnetic heating [28]. These stimuli-triggered strategies have great potential to be applied in fields other than bone cancer treatment.

In addition to stiff implants for bone cancer, implants were also produced with high flexibility for delicate internal organ drug delivery and wound-care applications [24]. For example, flexible patches have been fabricated to treat pancreatic cancer, and they have demonstrated good anticancer effectiveness and biodegradability in both in vitro and in vivo tests [34]. In addition to this, 3D-printed hydrogels are considered a promising dosage form for promoting cell proliferation and cell differentiation, offering physical support, drug-delivering and aiding cell regulating factors. Anticancer drug-loaded hydrogels with a solid disc shape made by extrusion-based printing are able to swell up by two-fold in water within 1 h and provide biphasic drug release for 24 h [35]. Another implantable hydrogel-based mesh loaded with temozolomide-release microparticles was formulated to prevent the recurrence of glioblastoma after resection surgery [36].

### 5.2. 3D Printing of Brachytherapy Devices

Three-dimension-printed brachytherapy applicators, compared to conventional applicators, have been demonstrated to provide personalised radiation exposure for targeted coverage while minimising unwanted exposure to avoid medical complications [24]. Reports of 3D-printed brachytherapy devices have increased significantly in the last decade. Kim et al. have demonstrated the use of customised applicators with good dose distribution and fixation for gynaecological cancer patients after surgery [37]. Jacob et al. have also demonstrated the development of a 3D-printed vaginal template for brachytherapy which can be used in cervical cancer [38]. Chmura et al. developed a superficial brachytherapy device using an SLA printer to treat skin cancer [39]. Other brachytherapy devices have also been developed for breast [40] and skin cancers [41,42].

### 5.3. 3D Printing of Local Immunotherapy Implant

Although the application of 3DP for cancer immunotherapy remains vacant, 3DP has possible applications in the field of cancer immunotherapy [43]. A 3D-printed nanogel implant releasing DNA nanocomplex was developed to eradicate residual glioblastoma cells post-surgery [44]. The implant was tested in a 3D-printed subcutaneous glioblastoma xenograft which significantly delayed the recurrence of glioblastoma. This study demonstrated the possibility of developing local gene therapy devices using 3DP technology. In the near future, 3DP could produce an artificial tertiary lymphoid which could be implanted to provide specialised immune cells for individual patients [43].

## 6. 3D Printing of Oral Solid Dosage Forms

With the combination of varying parameters such as printing ink compositions, tablet shapes, infill densities, many 3D-printed solid dosage forms have been produced with a variety of release kinetics, including immediate, sustained or delayed-release. These versatile release profiles made possible by 3DP provide many advantages over tablets made by traditional manufacturing, such as rapid prototyping and optimisation, improved bioavailability, better personalisation, ease of swallowing and multi-functions [24]. For instance, the first FDA-approved 3D-printed oral tablet, Spritam, which is of high porosity and dissolve within 11 sec, is aimed to resolve the difficulty in swallowing [45]. Recently, researchers have produced personalised oral tablets containing 5-FU using Drop-On-Powder 3DP technology. This tablet can be loaded with a personalised unit dose of 5-FU in high accuracy and shape fidelity [46].

3DP technology is also suitable to make “polypill”, which refers to a tablet containing several drugs. With 3DP, it is possible to combine incompatible APIs in a different compartment within a single pill. Polypills have been used in the treatment of cardiovascular disease and HIV infection. In the future, polypills might be an ideal formulation for cancer treatment which is potentially suitable to provide synergistic effects and decrease side effects [47].

## 7. 3D Printing of Transdermal Dosage Forms

Transdermal drug delivery is a great alternative to oral drug delivery. Drug delivery through the skin has advantages such as avoiding the liver’s first-pass metabolism, reducing pill burden and achieving good patient compliance [24]. It was estimated that each year, there are more than 1 billion transdermal patches produced globally [24]. Three-dimensional printing, as a technology manufacturing product with precise and versatile shape, enables the design and printing of transdermal patches that perfectly contour human anatomy, such as the nose [48,49]. Transdermal microneedles (MNs) have attracted much attention in recent years for their ability to create superficial pores in a painless manner on the skin and deliver small molecule drugs or big molecules such as proteins [24]. Several MNs drug delivery applications have been published for the treatment of skin cancer. For example, Uddin et al. fabricated metal microneedle coated with anticancer agents 5-FU, curcumin and cisplatin using inkjet printing, which showed good skin penetration and in vitro permeability [50] (Table 2).

## 8. 3D Bioprinting of Cancer Cell Models

### 8.1. 2D Model vs. Animal Model vs. 3D Model vs. 3D-Bioprinted Model

Two-dimensional models have been conventionally used for cancer research due to their affordability and simplicity [62], and they have contributed to numerous drug discoveries and developments. However, the majority of researches does not directly translate into clinical use. This is attributed to the fact that 2D cell culture does not recapitulate the in vivo tumour microenvironment of humans (Table 3) [62,63]. On the contrary, animal models are expensive, and species difference [62] has led to a discrepancy in gene expression, protein expression and soluble factors (cytokines, growth factor, etc.), which are important to study the cancer progression. Three-dimensional cell culture models have been developed to overcome the issues but bring about longer culture time, unsatisfactory reproducibility and higher cost [63]. Bioprinting utilises the 3DP technology to embed viable cells, biomaterials and growth factors by layers onto a scaffold to construct a 3D bio-printed model that closely resembles the actual tissue or organ [64]. Three-dimensional bio-printed cell models have been developed to mitigate this problem, and this technology benefits from lowering the cost in tandem with increasing the flexibility and complexity of structural design [65].

### 8.2. 3D Bioprinters

Numerous bioprinters are available on the market, while the rest only develop bio-printed products based on their bioprinters. One notable mention is the first commercial bioprinter, Organovo’s NovoGen MMX™ bioprinter, which is used to construct a human breast cancer model with a detailed in vivo microenvironment and provides a better insight into anticancer drug response [68]. However, Organovo does not sell its bioprinter, but rather it grants access to its technology through a partnership [69].

Another notable mention is CELLINK, the first bioink company in the world. The company also developed the world’s novel universal bioink that can be used in all 3D bioprinting systems regardless of cell types [70]. The company has a wide range of commercial bioprinters in addition to bioinks, and it has gained support from several industry leaders and bodies, including the Food and Drug Administration (FDA), Johnson & Johnson, Merck, Novartis, Roche, etc. [70].

Recently, 3D bioprinters started to gain attention for their application in hospitals globally. For example, Rastrum is a bioprinter that is developed by Inventia Life Science, which is adopted by Peter MacCallum Cancer Centre in Melbourne, Australia, and it opens up the possibility to print tumour cells of the patient to be tested in the laboratories in order to tailor drug treatment for different patients [71]. Besides, Bordeaux University Hospital in France adopted the full-colour and multi-material 3D printer, Stratasys^®^ J750 [72]. Such an application is the hospital’s attempt to increase the success rate of complex kidney tumour surgery, in tandem with achieving better surgical planning and patient understanding of the disease [72,73]. A case study reported that CSIRO, together with Anatomics, collaborated with St. Vincent Hospital in Melbourne to create a titanium heel implant using an Arcam 3D printer for a heel bone cancer patient and avoided his leg amputation [74]. Regardless, although 3D printers have been used in various hospitals, their oncological application remains fairly new and limited, but wider adoption is expected in the future.

## 9. 3D Bioprinting of Cancer Cell Model

### 9.1. Angiogenesis Model 

Continuous induction of angiogenesis is one of the six hallmarks of cancer [75]. This causes the premature growth of leaky and disordered blood vessels [75], which affects the drug delivery to the tissues compared to the healthy blood vessels [76]. Angiogenesis also represents a crucial step to provide sufficient nutrients and oxygen to allow further growth of tumours [77], in addition to eliminating cell waste [78]. 

Three-dimensional bioprinting of the cancer cell model can include the incorporation of vessels, which is previously unable to achieve with other conventional cancer cell models [79]. Such progress enables a more detailed investigation of the effects of drug delivery into cancerous tissues [77]. Three-dimension-printed leaky vessel models can also be used to test the delivery of an anticancer agent to the tumour as the vasculature surrounding tumour cells is well fenestrated due to uncontrolled cell growth. This enables adjustment of the particle size and dosage of drugs to deliver the active compound more efficiently to the target site, which reduces side effects and toxicity [76].

Sacrificial bioprinting is one of the most commonly used methods in bioprinting the tumour vasculature [77]. The process usually involves the moulding of the hydrogel matrix on a bio-printed sacrificial template, followed by removal of the template to form microchannels within the hydrogel matrix; subsequently, endothelial cells are transplanted and formed on microchannels to mimic the tumour vasculature [77,79]. Alternatively, microfluidic bioprinting and stereolithography are used to vascularise a cancer model [79].

Lee et al. 3D-bioprinted a glioma-vascular niche model that can be used to study cancer angiogenesis and the tumour microenvironment [80]. The vessel was constructed via sacrificial bioprinting with gelatine serving as the sacrificial template, followed by adjacently embedding the glioma stem cells (GSC) obtained from the patient into the collagen matrix with varying laminin concentrations [80]. The gelatine was removed and endothelialised with human umbilical vein endothelial cells (HUVECs) [80]. Subsequently, a pump was connected to begin the fluid perfusion [80]. Another method is seen to create the vascular channel before injecting the GSCs [80]. The result shown by Lee et al. suggests that this glioma-vascular niche model demonstrates angiogenesis within the brain tumour; therefore, the study of the interactions within the tumour microenvironment is possible [80].

### 9.2. Tumour Microenvironment

The tumour microenvironment plays a chief role in the regulation of cancer progression [81]. Alteration of the tumour microenvironment may contribute to an increase in chemoresistance [81]. Although the 2D model that is traditionally used as the in vitro cancer model has laid down the foundation for cancer studies for many years, it fails to mimic the complex and heterogeneous tumour microenvironment due to structural, mechanical and biochemical composition insufficiency [82]. Alternatively, animal models are unable to reproduce desired human clinical outcomes due to species differences [81] and also give rise to various ethical issues [82]. 

Advances in 3DP allow spatio-temporal control over cell–cell interactions, cell–matrix interactions and tumour–stromal cells distribution, hence enabling the creation of 3D tumour models that better mimic the exact in vivo tumour microenvironment and its heterogeneity [67]. This facilitates the study of disease progression and drug screening [83], which results in earlier diagnosis and better cancer treatment [76]. For example, studies have shown that capturing the tumour–stromal cells interaction is particularly important as such interaction plays a major role in drug chemoresistance [78]. Additionally, 3D-printed models with a better in vivo tumour microenvironment may mitigate the risks of failure and enables the identification of issue at an earlier stage of drug research and development [81].

Zhao et al. have fabricated an in vitro cervical tumour model by 3DP Hela cells (cervical cancer cells) together with gelatine, alginate and fibrinogen hydrogels using forced extrusion [84]. The tumour model exhibits greater cell proliferation, cellular spheroid formation, substantial MMP protein expression, higher chemoresistance against paclitaxel and more phenotypes compared to 2D cell culture, thus rendering the study of heterogeneous tumour microenvironment viable [84].

### 9.3. Metastasis

Cancer metastasis is responsible for 90% of cancer-related deaths [85]. Cancer metastasis occurs when the cancer cells migrate from the primary tumour site and enter the circulatory or lymphatic system before invading a new secondary site of tissue or organ. It is important to capture the heterogeneous tumour microenvironment in order to understand the cause of metastasis [67]. 

Issues associated with the current 3D models include the lack of vasculature and difficulty in tracing the recruitment of stromal cells [67]. These cause the identification and study of metastasis to be challenging. 

Three-dimensional printing represents a key method to recapitulate the heterogeneous tumour microenvironment [67]. It enables the accurate placement of key cells, including tumour cells, stromal cells and blood vessels [86]. A vascularised 3D-printed model provides a better insight into angiogenesis and metastatic cascades such as invasion, intravasation and extravasation [67]. Additionally, the metastatic 3D model built by Meng et al. demonstrates the ability for such a model to mimic the in vivo drug delivery, hence rendering anticancer drug screening possible [86].

A novel approach utilising 3DP technology (stereolithography) has been reported by Zhu et al. to develop matrices with various structural shapes to study bone metastasis due to breast cancer [19]. Two breast cancer cell types, MDA-MB-231 and MCF-7, were seeded onto the 3D-printed PEG/PEG-DA hydrogel bone matrix [19]. Hydroxyapatite (HA) nanoparticles were also included for the first time into a 3D scaffold to better mimic the bone matrix [19]. Mesenchymal stem cells (MSC) are also cultured together with MDA-MB-231 [19]. This study has shown that both breast cancer cell types show metastatic properties, with MDA-MB-231 exhibiting greater metastatic potential [19]. MSC was demonstrated to affect disease progression and alters cellular behaviour, causing more spheroidal cell formation [19]. The bone matrix with a small square has the highest cell count due to the greatest porosity [19]. The three-dimension-printed bone matrix reported here also proves that the tumour microenvironment significantly increases chemoresistance [19]. 

### 9.4. Tumour Spheroids

Tumour spheroid is the aggregation of tumour cells in 3D. Tumour spheroid is superior to 2D cell culture as it mimics the tumour microenvironment and the ability to capture HIF-1α due to hypoxia; hence, such a model is widely used in cancer research and drug development [87]. The construction of a large spheroid (>500 µm in diameter) exhibits more ideal properties that are similar to the actual tumour ranging from 0.5 mm^3^ to 1 mm^3^ in size [88]. A large spheroid is used as an analogy to study various tumour characteristics, which includes hypoxia [89]. Similar to a spheroid, tumour cells on the outer section have better access to oxygen when compared to those on the inner core (100 µm from tumour vessels) [88]. Consequently, hypoxia leads to the production of lactate, a decrease in pH and possibly cancer cell quiescent [88,89]. It is important to understand these characteristics obtained from tumour spheroids because they have a major impact on the therapeutic response of cancer cells to drugs [88]. 

Three-dimensional printing is one of the ways to create tumour spheroids, which provide advantages such as integration with imaging and biochemical assays [89]. Swaminathan et al. demonstrate the possibility to construct an entire 3D breast spheroid directly via bioprinting apart from bioprinting the individual breast cancer cells [90]. It shows for the first time that the former method facilitates the faster construction of functional tumour models while retaining the viability and structure of breast epithelial cells in different bioinks. When bioprinted in a co-culture system consisting of breast epithelial cells and endothelial cells, it can perform nearly instantaneous drug screening and other functional tests [90].

Liao et al. developed an innovative 3D-printed stamp-like resin mould to culture a tumour spheroid [91]. The aid of 3DP technology enables the fabrication of tumour spheroid with greater convenience, affordability, reusability, faster spheroid formation, better tumour size control, minimal cell consumption, easy medium exchange and improved batch consistency [91]. Hence, Liao et al. successfully demonstrate that their 3DP application may permit high-throughput anticancer drug screening and personalised treatment [91]. It is also seen that 3DP application on tumour spheroid formation is superior to the conventional hanging drop method and ultralow attachment culture plate [91].

### 9.5. Organs-On-Chips: Microfluidics System

Organs-on-chips are designed to mimic actual human organ functions by fabricating cells along with chambers and channels into a microfluidic device [92]. Three-dimensional printing is seen as an automated, efficient and cost-efficient method to produce organs-on-chips [93]. It allows the fabrication of complex channels, tissues and heterogenous structures with greater heterogeneity that closely resembles the human physiological organ functions [93]; thus, it may serve as a drug screening platform [92]. 

There are three ways in which organs-on-chips can be created using 3DP technology. (i) First, 3DP technology contributes to the development of unibody microfluidic devices, which in turn allows greater design flexibility, a simpler manufacturing process of microfluidic devices and easier integration of input and output interfaces [94]. (ii) Another way is the 3D bioprinting of tissues/structures on microfluidic devices, and it is a two-step fabrication process that enables the construction of dynamic and heterogenous tissues or organs on the pre-fabricated perfusable chip [92]. (iii) Alternatively, the one-step fabrication, which is the 3DP of the entire organ-on-a-chip [92], demonstrates a more favourable characteristic in terms of automation, strength and efficiency; however, the accuracy and transparency aspects require further improvements [94]. 

In the context of cancer, a tumour-on-a-chip can be created based on the microfluidics system [87]. The integration of 3DP technology and microfluidics system enhances the structural details of a tumour model [95], as cellular scaffolds with better resolution and porosity can be fabricated [96]. Three-dimensional printing also facilitates the fabrication of multiple cancer cell types directly into the microfluidic platform, thereby creating a biomimetic environment for high-throughput testing [97]. Microfluidic devices hold key advantages over static culture; in particular, mechanical features such as fluid flowing in and out of cells, causing shear stress, plays a major role in recapitulating the in vivo microenvironment [87]. This concept of shear stress has also been reported as a cause of cell cycle arrest in cancer cells [87]. Additionally, it is proven that cancer cells migrate along the direction of the fluid stream in 3D scaffolds [95]. Microfluidic systems are able to manipulate various flow patterns to generate different chemical concentrations, which has the possibility to enhance further studies in cancer metastasis [95]. Tumour-on-a-chip can be customised, and permits live data tracking; therefore, the possibility to capture circulating tumour cells (CTC) and drug screenings are plausible [89].

Chen et al. described a microfluidic device capable of isolating circulating tumour cells (CTC) from peripheral blood samples [98]. This device is constructed using the ProJet 3000HD 3D printer with an inner core embedded with anti-EpCAM antibodies [98]. The 3D-printed microfluidic devices are favourable to those that are based on the conventional PDMS or thermal plastic substrate [98]. Therefore, their microfluidic device shows the higher surface area and fluid stream manipulation, which facilities the anti-EpCAM antibodies assisted capturing positive tumour cell lines such as MCF-7 breast cancer, SW480 colon cancer and PC3 prostate cancer at a high efficiency rate [98]. Overall, the performance of this 3D-printed microfluidic device may drive the advancement in cancer diagnosis, metastasis detection and cancer treatment [98]. 

### 9.6. 3D Bioprinting for Anticancer Drug Development and Therapeutic Screening

Cancer drug development poses a challenging task as only 5% of the drugs successfully transition into the market [99] and costs approximately 800 million USD [100]. This might be attributed to the fact that 2D cultures and animal models do not recapitulate the in vivo tumour microenvironment, unlike the 3D-printed cancer models [2], in which the latter also display greater drug resistance [101]. In recent years, there has been a surge in researches using 3DP technology for drug development. For instance, Chen et al. developed a novel 3D-printed microfluidic system that is capable of combining various cancer drugs and potentially increases the effectiveness of cancer treatment [65]. Additionally, the aforementioned microfluidic chip created is able to achieve better scalability, accuracy and is more compact, which is largely attributed to the 3DP capability to fabricate complex and flexible design [65].

A 3D-printed anticancer drug screening model has been constructed by Zhao et al. with gelatine, alginate and fibrinogen serving as the matrix [102]. This study has adopted hepatocyte or/and adipose-derived stem cells (ADSC) as the subject to evaluate drug screening in 2D and 3D-printed models [102]. Various stains and three different drugs, including 5-FU, astragalus polysaccharide (AP) and matrine were used in three sets with different concentrations [102]. Gelatine with high and low concentrations has also been alternated in the matrix [102]. Based on the study conducted by Zhao et al., it has shown that the 3D model demonstrates a greater connection between cells as cells migrate to the extracellular matrix similar to the tumour microenvironment in vivo [102]. It is also shown that a low concentration of gelatine facilitates more cell–cell connections in the 3D-printed model [102]. Anticancer drug concentration also has a significant impact on cell survival and drug resistance; for instance, 5-FU is more effective in inhibiting cell survival in low concentrations while high concentrations lead to a rebound; also, co-culture of hepatocyte/ADSC exhibits the greatest drug resistance [102]. In comparison to 2D cell culture, a model created with 3DP technology is more likely to enable high-throughput, scalable and reliable drug screening [102].

## 10. The Limitation of 3D-Bioprinted Cancer Models

There are several limitations of 3D-bioprinted models (Table 4). One challenge is the inconsistency in drug responses from different 3D printing methods [45], which is further hindered by the limited choices of bioinks and biomaterials along with diverse bioprinters specifications [69]. This proves that a more streamlined drug screening result and bioprinting process are left to be desired [45,80]. The selection of appropriate bioinks is limited, but it is extremely important in bioprinting. Aspects of bioinks such as transparency, biocompatibility, viscosity, photo-curability and crosslink ability must all be considered based on the type of bioprinters [68,80,94]. The resolution, scalability, accuracy, printing speed and reproducibility of 3D bioprinters remains a challenge, where there are no current bioprinters that excel in all aspects. Currently, the majority of the 3D bio-printed models are scaled-down; thus, bioprinting an actual size tumour model is a challenge to tackle in the future [68,80]. Although there are 3D models that are bioprinted with vasculature, tumour microenvironment or metastatic progression, it still lacks a 3D-bioprinted model that is completely incorporated with every criterion that enables the detailed cancer research.

Looking at 3DP technology, it is an evolving field with promising reported results. However, attempts to standardise the printing process should be considered to reduce bias. Despite the fact that some studies were reported [103,104] to prove the accuracy of the models compared to the actual organs, more extensive studies should be conducted to avoid any surgical errors and encourage the use of the technology. Finally, there is still a chance for improvement when it comes to printing materials to provide more realistic models.

## 11. 3D Printing of Nonbiological Medical Devices

### 11.1. 3D-Printed Models for Training and Planning of Cancer-Related Procedures

Surgical training is usually carried out on cadavers which present realistic models to an extent; however, there are constraints to their use, namely, the availability of dead bodies, high cost of dissection labs maintenance, safety concerns regarding prolonged contact with the bodies and preservation materials and ethics [105]. Also, cadavers do not mimic varied pathological conditions of individual surgical cases such as blood flow, which should be considered during surgery. Surgery is an essential strategy in many treatments and palliative protocols for cancer worldwide [106]. Operations such as tumour resection or replacement of the diseased organ with a donated organ are used. Cancer surgeries involve complex training compared to other procedures, which presents an issue due to restricted working hours and shorter training programs for young surgeons [107]. Consequently, the need for models for effective training has been provided by 3DP models representing different human anatomies and malignancies. Models could be created based on patients’ images, thus demonstrating individualised models that also act as a useful tool in patient counselling in addition to training and planning purposes [108,109]. Giovanni et al. [110] displayed 3D-printed models revealed by the literature for different purposes in urology, namely, kidney, prostate, ureter, adrenal gland, iliac vessels and bladder models. Table 5 demonstrates some 3D-printed models used in oncology.

An organ transplant may be necessary in cases where cancer has rendered an organ ineffective, as in liver cancer [111]. Three-dimension-printed models of the donor organ and recipient cavity could be created for planning the procedure and predicting the suitability of the transplanted organ anatomically, thus avoiding unnecessary surgery [112].

In addition, determination of the exact tumour size before surgery is essential for successful resection and prevention of recurrence [113]. Currently, 3D imaging is an important tool for planning cancer surgeries; however, the images are presented on 2D computer screens, which do not provide the same detail as the 3DP models offer [114,115]. 

Furthermore, the presence of a physical replica during operations aids surgeons to accurately navigate through critical areas. This is best demonstrated in procedures that include the urological system where miniaturised medical tools are used [116] and paediatric surgery where challenges arise such as smaller body cavities and more delicate tissues as opposed to adults [117]. 

Additionally, 3D models can be positioned in any way to mimic the actual orientation of the organ inside the body; for example, patients are placed in the flank position during renal surgery, exposing the kidney in a different rotation from preoperative images [118]. 

### 11.2. 3D Printing of Prosthetics after Tumour Surgery

Head and neck cancer surgery involve the removal of a significant portion of facial structures, rendering the patient deformed with loss of partial or complete function of buccofacial features, which requires reconstructive surgery [119]. Exact 3D models featuring the patient’s anatomy improves preoperative planning, intraoperative navigation and shortens the duration of surgery. Traditionally, bone grafts are used; however, they are not optimal due to limited availability, risk of wound infection and the possibility of resorption [120]. A research group in Brazil reconstructed a patient’s face, who had lost her eye and part of her jaw as a result of cancer, using 3DP. Images were simply taken by a smartphone and utilised to create protheses matching the patient’s facial features. The process was reported to be fast (12 h), cost-effective (silicon, resin and synthetic fibres) and less invasive (sculptures from manual facial imprints were replaced by digital facial impressions) [121,122]. Likewise, another cancer patient had his speech and eating habits restored after using 3DP prosthetics [123]. Herein, customised implants exhibiting high accuracy and showcasing complex structures can be designed and printed to act as alternatives to grafts in orbital reconstruction [124,125], craniofacial and maxillofacial implants [126,127,128,129], mandibular contouring and reconstruction [130,131] and nasal reconstruction [132,133].

Conventional breast implants used by cancer survivors who have undergone mastectomy deteriorate with time and need to be replaced, adding a financial burden on the patient. myReflection^®^ has introduced a breast implant based on the 3DP concept, made of elastic, stable and tear-resistant materials which promise to last for a longer time and be cost-effective in the long run [134]. Likewise, breast implants were fabricated via 3DP for Poland syndrome patients [135].

In 2014, the first 3DP vertebrae were successfully implanted in a child suffering from a tumour in the spinal cord. Traditional implants are fixated using screws or orthopaedic cement; however, since 3DP allows the production of any shape, the printed vertebrae were aligned perfectly with the surrounding bones without the need for fixation [136]. 

An Italian hospital adopted 3D technology to produce titanium bone implants for patients with osteosarcoma, where the replacement of the diseased bone was required. It was found that the new implants posed a lower infection risk, allowed speedy patient recovery and provided a better alternative to traditional prostheses [137]. The same idea was also considered by other research groups helping many patients around the world [138,139].

Moreover, 3DP has played a pronounced role in prostheses manufacture of limbs, especially for pediatric use where the child’s growth requires a continuous change of the prosthetic limb, which is expensive [140].

Moving on to diagnosis, prostate cancers are usually diagnosed by transrectal ultrasound-guided biopsy. However, incidents of missed cancers have been noted [141]. Three-dimensional models fabricated from the patient’s magnetic resonance images and printed using transparent resin display the shape, size and location of the tumour, enabling the physicians to decide on the best sampling strategy [142].

### 11.3. Limitations of 3D-Printed Nonbiological Medical Devices

Although 3DP has proved to be advantageous in many aspects, it has its flaws. Three-dimension-printed models that exactly resemble human tissues and organs do not exist to date [143]. Human organs and vasculature are more flexible and softer than some of the printing materials used to fabricate the 3D models. In a kidney model created for adrenalectomy training for neuroblastoma, vessels and tumours were hard to excise due to the hardness of the printing material; also, fibrous adhesions associated with preoperative chemotherapy were not featured [144]. Nevertheless, other printing materials are currently available such as TangoPlus^®^ and VeroClear, which are flexible, imitating the texture of human anatomy. They are also available in different colours and transparencies to allow the fabrication of realistic models illustrating vasculature with improved clarity. That being said, these materials are expensive and significantly add to the cost of the model.

As 3DP is considerably a modern technique, there are no unified imaging protocols, printing materials, printers and software used, which results in different outcomes [145], so tumour size or extent of invasion might differ in reality.

In addition, time is of the essence in cancer treatment to prevent metastasis and further tumour growth, fast surgical intervention and thus planning is required, which could be problematic as the production of a model takes time. The process of 3DP includes imaging, segmentation and surface modelling, model processing, printing [146] and colouring when monochrome printers are used [147]. The literature revealed processing times from 25 h [115] to 4 working days [148].

Besides, the cost of the technology is variable and depends on several factors such as the quantity, printing materials and type of printer. These financial considerations may limit the use of 3DP in oncology and training programs. Expenses are expected to drop as the 3DP technology prevails and demand rises [109].

**Table 5:** Examples of some 3D-printed models for organs.

Organ	Purpose	Printing Material	Reference
Kidney	Surgical planning and patient counselling of a partial nephrectomy procedure	Thermoplastics	[147]
Liver, Kidney, Lung, Prostate and Arteries	Medical education	Polyamide	[149]
Liver	Liver transplant procedure	TangoPlus, VeroClear, TangoBlack and VeroBlue	[115]
Liver	Medical education	Nylon	[150]
Liver	Surgical planning for hepatectomy for colorectal cancer metastases	Silicon	[151]
Liver	Pre-operative planning	TangoPlus and TangoBlack	[152]
Bladder and Urethra	Robotic vesicourethral anastomosis	Silicon	[40]
Brain	Surgical planning in pediatric glioma	VeroClear	[153]
Brain	Neurosurgical planning	-	[154]
Mandible	Mandibular reconstruction	Photopolymer	[155]
Lung	Patient counselling in stage I cancer	Photopolymer	[156]
Thorax	Surgical planning for removal of the thoracic tumour	-	[153]

## 12. Challenges and Future Orientations

There are technical and regulatory challenges and limitations as 3DP technology is still relatively new in oncological applications. The material used must be biocompatible to meet the effectiveness and safety requirements of human usage and consumption [24]. Not all printable materials are biocompatible; even though the large molecular weight polymers are compatible, the risk of monomers leaching still exists, and the heating or laser sintering printing process might cause drug degradation, which brings great safety concerns [157]. Although 3DP can be performed in an aseptic environment, sterilisation is often required for the final product. However, many 3DP materials, such as polymers, have limited choices of sterilization, and the stability of drugs under heat and light should also be considered [24]. These safety concerns have hindered regulatory approval and lead to a low clinical trial rate of 3D-printed medicine. Traditional clinical trials often require a certain number of patients, varying from 20 to 3000 according to the phase of the clinical trial. However, because many 3D-printed products are tailored for individual patients, the difficulty of meeting the requirement of the FDA via the traditional approval route has impeded the introduction of 3D-printed pharmaceutics to the market [158].

The potential of 3DP for cancer applications remains to be exploited. Three-dimensional printing could bring revolution to traditional pharmaceutical industries and current medical systems by its potential to produce a biocompatible and functioning product such as 3D-printed personal organs, cancer and surgical models and customised multifunctional medicine, which is promising in terms of reducing R&D cost and duration, providing quick feedback from individual patients and achieving the ultimate goal of personalisation.

## Figures and Tables

**Table 1:** 3DP in the applications of cancer according to the type of cancer.

Type of Cancer	Number of Papers Mentioned	Percentage (%)
Non-specific	69	25.4%
Breast	40	14.7%
Brain	22	8.1%
Bone	21	7.7%
Head and Neck	16	5.9%
Gynaecological	15	5.5%
Kidney	13	4.8%
Lung	11	4.0%
Prostate	11	4.0%
Colorectal	10	3.7%
Liver	9	3.3%
Skin	9	3.3%
Pelvic	5	1.8%
Pancreatic	4	1.5%
Spinal	3	1.1%
Thoracic	3	1.1%
Bladder	2	0.7%
Thyroid	2	0.7%
Bile duct	1	0.4%
Cartilage	1	0.4%
Chest wall	1	0.4%
Chondrosarcoma	1	0.4%
Intestinal	1	0.4%
Mandible	1	0.4%
Sternal	1	0.4%
**TOTAL**	272	100.0%

**Table 2:** 3DP of anticancer dosage forms.

Types of Dosage Forms	Dosage Forms	APIs	Diseases	Types of Printer	Matrixes	References
Implants for local chemotherapy or thermotherapy	Scaffold	DOX	Bone cancer	FDM	Chitosan, nanoclay and β-tricalcium phosphate, PCL	[27]
	Drug-eluting implant	DOX and apo2l/trail	Bone cancer	SLM	Ti6A14V	[51]
	Magnetic hyperthermia scaffold	DOX	Bone cancer	PE	Fe_3_O_4_/MBG/PCL	[28]
	Photothermal scaffold	non	Bone cancer	N/A	Ca-P/polydopamine	[29]
	Photothermal bioscaffold	non	Bone cancer	N/A	Fe-CaSiO_3_	[30]
	Photothermal hydrogel scaffolds	PDA	Bone cancer	Bioscaffolder	Alg-PDA	[31]
	Nanoporous disc	DOX	Bone metastases secondary to prostate cancer	FDM	TPU	[52]
	Tablet	Progesterone	Breast, ovarian, uterus and prostate cancers	SLS	PCL	[53]
	Bullet-shaped implant	Cytoxan	N/A	FDM	PLA	[54]
	Magnetically actuated implant	Methylene blue (MB), Docetaxel (DTX)	Prostate cancer	N/A	ABS	[32]
	Magnetically controlled implant	TNF-related apoptosis-inducing ligand (TRAIL) and DOX	N/A	Bioprinter	graphene oxide and PCL composite	[33]
	Scaffold	DOX and Cisplatin	Breast cancer	E-jet	PLGA	[55]
	Scaffold	5-FU and NVP-BEZ235	Breast cancer	E-jet	PLGA	[55]
	Spherical implant	DOX, ifosfamide, methotrexate, Cisplatin (CDDP)	Osteosarcoma	SLA	PLLA	[56]
	Patch	5-FU	Pancreatic cancer	PE, MHDS	PLGA, PCL	[34]
	Tablet	Fluorouracil	Cartilage cancer	SLS	PCL	[57]
	Drug delivery implant patent	N/A	Mouth/anal/cervical/vaginal cancer	N/A	N/A	[58]
	Nanogel discs	Paclitaxel, rapamycin	Ovarian cancer	FDM	Poloxamer 407	[35]
	Mesh	Temozolomide (TMZ)	Glioblastoma (GBM)	Bioprinter	PLGA	[36]
Brachytherapy device	Vaginal template for brachytherapy	N/A	Cervical cancer	Multi-jet Printing	N/A	[38]
	Superficial brachytherapy applicator	Radioisotopes of yttrium-90	Skin cancer	SLA	PLA	[39]
	Brachytherapy applicator	Gafchromic ebt3 film	Gynaecologic cancer	FDM	PLA	[37]
Implants for local Immunotherapy	Nanogel	DNA nanocomplex	Glioblastoma	SLA	Gelatin Methacrylamide	[44]
Transdermal Dosage forms	Anticancer agent coated metal microneedle	5-fluorouracil, CUR, cisplatin	Skin cancer	MJ	Metal	[50]
	Microneedle	Decarbazine	Skin cancer	SLA	Propylene fumarate (PPF)/diethyl fumarate (DEF)	[59]
	Microneedle	Cisplatin	Skin cancer	SLA, inkjet printer	Soluplus^®^	[60]
Oral dosage forms	Tablet	5-fluorouracil	Colorectal cancer	DOP	Caso4, Soluplus^®^	[46]
Not stated	Microparticles	Paclitaxel (PTX)	Cervical Cancer	Piezoelectric inkjet printer	PLGA	[61]

Abbreviations: APIs: active pharmaceutical ingredients, Ca-P: calcium phosphate, CUR: curcumin, DOX: Doxorubicin, 5-FU: Fluorouracil, FDM: fused deposition modelling, SLM: selective laser melting; PDA: poly dopamine, PE: pneumatic extrusion, SLS: selective laser sintering, MHDS: multi-head deposition system, SLA: stereolithographic, MJ: material jetting, DOP: digital offset press technology. PTX: paclitaxel, PLGA: poly (lactic-co-glycolic acid), PLA: polylactic acid, PCL: polycaprolactone, PLLA: poly (l-lactic acid), ABS: acrylonitrile butadiene styrene, TNF: Tumour necrosis factor, TPU: thermoplastic. Polyurethane, ALG-PDA: sodium alginate/poly dopamine, MBG: mesoporous bioactive glass, NVP-BEZ235: dactolisib. E-jet: electrohydrodynamic jet, N/A: not applicable.

**Table 3:** Advantages and disadvantages of 3D-bioprinted cancer models compared to other models.

Models	Advantages	Disadvantages	References
2D culture	Good reproducibility Low cost Easy to culture	Lack of cell–cell and cell–extracellular interaction Fails to mimic in vivo tumour microenvironment Loss of various phenotypes	[62,63]
Animal (mouse) model	Short lifespan Less genetic variations Plenty of genetic information	Expensive Homozygosity Unreliable predictions for drug safety and efficacy Different responses to certain gene expression Different organ systems Ethical issues	[21,66]
3D cell culture model	Presence of cell–cell and cell–extracellular interactions Mimics in vivo tumour microenvironment Various phenotypes are maintained In vivo gene expressions are maintained	Long culture time Can have bad reproducibility More expensive than 2D cultures	[63]
3D bio-printed cell model	Low cost Able to fabricate complex structures Presence of cell–cell and cell–extracellular interactions Better in mimicking in vivo tumour microenvironment	Limited choice of materials is important depending on type of 3D printer Low resolution for certain types of 3D printer Low printing speed	[63,67]

**Table 4:** Examples of 3D-bioprinted cancer cell models.

Model	Tumour Type (Cell line)	Matrix	Drug	Type of Printer	Features	References
Glioma-vascular niche	Brain cancer (Glioblastoma multiforme)	Collagen type with laminin	N/A	N/A	Angiogenesis, Cell–cell/Cell–ECM interaction	[81]
In vitro Cell Laden	Cervical cancer (cell line HeLa)	Gelatine, alginate, fibrinogen	Paclitaxel	N/A	Drug toxicity study	[84]
3D bone matrix	Breast cancer (cell line MDA-MB-231, MCF-7, MSC)	PEG PEG-DA HA	N/A	Stereolithography	Metastatic study	[19]
3D Microfluidic device	MCF-7 breast cancer, SW480 colon cancer, (cell line PC3) prostate cancer	N/A	Anti-EpCAM antibodies	Multi-jet printing	CTC isolation	[98]
3D Drug screening	Hepatocyte/ADSC	Gelatine, alginate, fibrinogen	5-FU, AP, matrine	N/A	Drug screening	[102]

Abbreviations: 5-FU: 5-fluorouracil, ADSC: adipose-derived stem cells, AP: astragalus polysaccharide, CTC: circulating tumour cells, ECM: extracellular matrix, EpCAM: epithelial cell adhesion molecule, HA: hydroxyapatite, MSC: mesenchymal stem cell, PEG: polyethylene glycol, PEG-DA: polyethylene glycol diacrylate.

## Data Availability

Data sharing is not applicable.

## Supplementary Materials

The following are available online at https://www.mdpi.com/article/10.3390/ph14080787/s1, Table S1: Cancer research using 3D printing technologies according to year of publication, Table S2. Clinical trials related to cancer utilising 3D printing technologies by country, Table S3. Patent of 3D printing technologies for cancer applications according to year of publication.
