# Supplementary material for: Three-Dimensional Printing for Cancer Applications: Research Landscape and Technologies
Source: Pharmaceuticals (Basel). 2021 Aug 10;14(8):787. doi: 10.3390/ph14080787 (PMC8401566; doi:10.3390/ph14080787)
Supplement: Supplementary file 1 [file pharmaceuticals-14-00787-s001.zip › pharmaceuticals-1328335-supplementary.pdf]

Supplementary Information

Table S1. Cancer research using 3D printing technologies according to year of publication.

Year	Title	Tumour	References
2009	Mandibular reconstruction using stereolithographic 3-dimensional printing modeling technology	Mandible	[1]
2011	A three-dimensional in vitro ovarian cancer coculture model using a high-throughput cell patterning platform	Gynaecological	[2]
2012	3-D printout of a DICOM file to aid surgical planning in a 6-year-old patient with a large scapular osteochondroma complicating congenital diaphyseal aclasia	Bone	[3]
2012	Fabrication of drug-loaded polymer microparticles with arbitrary geometries using a piezoelectric inkjet printing system	Non-specific	[4]
2012	Fabrication and characterization of a rapid prototyped tissue engineering scaffold with embedded multicomponent matrix for controlled drug release	Bone	[5]
2013	Preoperative Three-Dimensional Model Creation of Magnetic Resonance Brain Images as a Tool to Assist Neurosurgical Planning.	Brain	[6]
2013	3D volumetric analysis for planning breast reconstructive surgery	Breast	[7]
2014	A Low-Cost Surgical Application of Additive Fabrication. Journal of Surgical Education	Liver	[8]
2014	Three-dimensional print of a liver for preoperative planning in living donor liver transplantation	Liver	[9]
2014	3D-printed magnetic Fe 3 O 4/MBG/PCL composite scaffolds with multifunctionality of bone regeneration, local anticancer drug delivery and hyperthermia	Bone	[10]
2014	Three-dimensional printing of Hela cells for cervical tumor model in vitro	Gynaecological	[11]
2015	3D functional and perfusable microvascular networks for organotypic microfluidic models	Non-specific	[12]
2015	Anti-Cancer Drug Screening Based on a Adipose-Derived Stem Cell/Hepatocyte 3D Printing Technique	Non-specific	[13]
2015	Generation of 3-D glioblastoma-vascular niche using 3-D bioprinting	Brain	[14]
2015	Microstereolithography and characterization of poly (propylene fumarate)-based drug-loaded microneedle arrays	Skin	[15]
2015	Inkjet printing of transdermal microneedles for the delivery of anticancer agents	Skin	[16]
2015	Magnetically controlled nano-composite based 3D printed cell scaffolds as targeted drug delivery systems for cancer therapy	Bone	[17]
2015	A cylindrical magnetically-actuated drug delivery device proposed for minimally invasive treatment of prostate cancer	Prostate	[18]
2015	3D-printing microfludic device for breast cancer cell culture and pharmacological research	Breast	[19]
2015	Bioprinting for cancer research	Non-specific	[20]
2015	Individualized physical 3-dimensional kidney tumor models constructed from 3-dimensional printers result in improved trainee anatomic understanding	Kidney	[21]
2015	Non-small cell lung cancer 95D cells co-cultured with 3D-bioprinted scaffold to construct a lung cancer model in vitro	Lung	[22]
2015	Three-dimensional model for surgical planning in resection of thoracic tumors	Thoracic	[23]

2015	Three-dimensional printing technique assisted cognitive fusion in targeted prostate biopsy	Prostate	[24]
2015	Tridimensional titanium-printed custom-made prosthesis for sternocostal reconstruction	Bone	[25]
2016	3D bioprinted glioma stem cells for brain tumor model and applications of drug susceptibility	Brain	[26]
2016	Understanding Spatially Complex Segmental and Branch Anatomy Using 3D Printing: Liver, Lung, Prostate, Coronary Arteries, and Circle of Willis	Non-specific	[27]
2016	3D printed nanocomposite matrix for the study of breast cancer bone metastasis	Breast	[28]
2016	3D Printed Tissue Models: Present and Future	Non-specific	[29]
2016	3D printing of biomaterials with mussel-inspired nanostructures for tumor therapy and tissue regeneration	Bone	[30]
2016	A 3D-printed local drug delivery patch for pancreatic cancer growth suppression	Pancreatic	[31]
2016	A Nanoprinted Model of Interstitial Cancer Migration Reveals a Link between Cell Deformability and Proliferation	Intestinal	[32]
2016	A patient-specific polylactic acid bolus made by a 3D printer for breast cancer radiation therapy	Breast	[33]
2016	Application areas of 3D bioprinting	Non-specific	[34]
2016	Application of anatomically accurate, patient-specific 3D printed models from MRI data in urological oncology	Kidney<br>Prostate	[35]
2016	Bioprinting the Cancer Microenvironment	Non-specific	[36]
2016	Cancer drug discovery: recent innovative approaches to tumor modeling	Non-specific	[37]
2016	Clinical implementation of 3D printing in the construction of patient specific bolus for electron beam radiotherapy for non-melanoma skin cancer	Skin	[38]
2016	CT guidance 125I seed implantation for pelvic recurrent rectal cancer assisted by 3D printing individual non-coplanar template	Rectal	[39]
2016	Evaluation of three-dimensional printing for laparoscopic partial nephrectomy of renal tumors: a preliminary report	Kidney	[40]
2016	Image-guided installation of 3D-printed patient-specific implant and its application in pelvic tumor resection and reconstruction surgery	Pelvic	[41]
2016	Individualised 3D printed vaginal template for MRI guided brachytherapy in locally advanced cervical cancer	Gynaecological	[42]
2016	Irreversible Electroporation of Prostate Cancer: Patient-Specific Pretreatment Simulation by Electric Field Measurement in a 3D Bioprinted Textured Prostate Cancer Model to Achieve Optimal Electroporation Parameters for Image-Guided Focal Ablation	Prostate	[43]
2016	Personalized 3D printed model of kidney and tumor anatomy: a useful tool for patient education	Kidney	[44]
2016	Reproducing 2D breast mammography images with 3D printed phantoms	Breast	[45]
2016	Three-dimensional culture systems in cancer research: Focus on tumor spheroid model	Non-specfic	[46]
2016	Tumor resection at the pelvis using three-dimensional planning and patient-specific instruments: A case series	Pelvic	[47]
2017	3D bioprinting: Improving in vitro models of metastasis with heterogeneous tumor microenvironments	Non-specific	[48]
2017	3D printing of PCL/Fluorouracil tablets by selective laser sintering: Properties of implantable drug delivery for cartilage cancer treatment	Cartilage	[49]
2017	Laser Printing of PCL/Progesterone Tablets for Drug Delivery Applications in Hormone Cancer Therapy	Breast	[50]

2017	Engineering of micro-to nanostructured 3d-printed drug-releasing titanium implants for enhanced osseointegration and localized delivery of anticancer drugs	Non-specific	[51]
2017	3D-Engineered Conformal Implant Releases DNA Nanocomplexs for Eradicating the Postsurgery Residual Glioblastoma	Brain	[52]
2017	Development of 3D printed applicator in brachytherapy for gynecologic cancer	Gynecologic	[53]
2017	3D printed pathological sectioning boxes to facilitate radiological-pathological correlation in hepatectomy cases	Liver	[54]
2017	3D printed renal cancer models derived from MRI data: application in pre-surgical planning	Kidney	[55]
2017	3D printing for the development of in vitro cancer models	Non-specific	[56]
2017	A Patient-Specific 3D-Printed Form Accurately Transfers Supine MRI-Derived Tumor Localization Information to Guide Breast-Conserving Surgery	Breast	[57]
2017	A three-dimensional pelvic model made with a three-dimensional printer: applications for laparoscopic surgery to treat rectal cancer	Colorectal	[58]
2017	Acoustic and hybrid 3D-printed electrochemical biosensors for the real-time immunodetection of liver cancer cells (HepG2)	Liver	[59]
2017	Application of 3D printing in urology	Kidney<br>Prostate	[60]
2017	Application of 3D soft print models of the kidney for treatment of patients with localized cancer of the kidney (a pilot study)	Kidney	[61]
2017	Application value of 3D printing technology in the surgery of sphenoid ridge meningioma	Brain	[62]
2017	Automated 3D-printed unibody immunoarray for chemiluminescence detection of cancer biomarker proteins	Non-specific	[63]
2017	Bioprinting of three-dimensional tumor models: A preliminary study using a low cost 3D printer	Non-specific	[64]
2017	Clinical applications of 3-dimensional printing in radiation therapy	Non-specific	[65]
2017	Coaxial 3D bioprinting of self-assembled multicellular heterogeneous tumor fibers	Brain	[66]
2017	Cost-effective, personalized, 3D-printed liver model for preoperative planning before laparoscopic liver hemihepatectomy for colorectal cancer metastases	Colorectal	[67]
2017	Design and fabrication of a 3D-printed oral stent for head and neck radiotherapy from routine diagnostic imaging	Head & Neck	[68]
2017	Dosimetry verification of radioactive seed implantation for malignant tumors assisted by 3D printing individual templates and CT guidance	Non-specific	[69]
2017	Engineering 3D Models of Tumors and Bone to Understand Tumor-Induced Bone Disease and Improve Treatments	Bone	[70]
2017	Engineering of Micro- to Nanostructured 3D-Printed Drug-Releasing Titanium Implants for Enhanced Osseointegration and Localized Delivery of Anticancer Drugs	Bone	[71]
2017	Evaluation of deformable image registration between external beam radiotherapy and HDR brachytherapy for cervical cancer with a 3D-printed deformable pelvis phantom	Gynaecological	[72]
2017	Evaluation of pre-surgical models for uterine surgery by use of three-dimensional printing and mold casting	Gynaecological	[73]
2017	Individualized 3D scanning and printing for non-melanoma skin cancer brachytherapy: A financial study for its integration into clinical workflow	Skin	[74]
2017	Introduction of novel 3D-printed superficial applicators for high-dose-rate skin brachytherapy	Non-specific	[75]
2017	Mandibular reconstruction after cancer: an in-house approach to manufacturing cutting guides	Head & Neck	[76]

2017	Multilevel 3D Printing Implant for Reconstructing Cervical Spine with Metastatic Papillary Thyroid Carcinoma	Thyroid	[77]
2017	One-step reconstruction with a 3D-printed, custom-made prosthesis after total en bloc sacrectomy: a technical note	Spinal	[78]
2017	Preliminary application of 3D printing coplanar template in treating pancreatic cancer with 125I seed implantation	Pancreatic	[79]
2017	Reconstruction of Thoracic Spine Using a Personalized 3D-Printed Vertebral Body in Adolescent with T9 Primary Bone Tumor	Bone	[80]
2017	Reconstruction with 3D-printed pelvic endoprostheses after resection of a pelvic tumour	Pelvic	[81]
2017	Semi-automated delineation of breast cancer tumors and subsequent materialization using three-dimensional printing (rapid prototyping)	Breast	[82]
2017	Therapeutic response assessment using 3D ultrasound for hepatic metastasis from colorectal cancer: Application of a personalized, 3D-printed tumor model using CT images	Colorectal	[83]
2017	Three-Dimensional Printing of a Hemorrhagic Cervical Cancer Model for Postgraduate Gynecological Training	Gynaecological	[84]
2017	Usefulness of three-dimensional modeling in surgical planning, resident training, and patient education	Pancreatic	[85]
2017	Utility and reproducibility of 3-dimensional printed models in pre-operative planning of complex thoracic tumors	Thoracic	[86]
2018	3D Bioprinting and Stem Cells	Breast	[87]
2018	Nanoporous 3D-Printed Scaffolds for Local Doxorubicin Delivery in Bone Metastases Secondary to Prostate Cancer	Prostate	[88]
2018	3D printing of high-strength bioscaffolds for the synergistic treatment of bone cancer	Bone	[89]
2018	3D bioprinting of functional tissue models for personalized drug screening and in vitro disease modeling	Non-specific	[90]
2018	3D printed microfluidic chip for multiple anticancer drug combinations	Non-specific	[91]
2018	3D printed models in mandibular reconstruction with bony free flaps	Head & Neck	[92]
2018	3D printed tissue engineered model for bone invasion of oral cancer	Head & Neck	[93]
2018	3D printing and coating to fabricate a hollow bullet-shaped implant with porous surface for controlled cytoxan release	Non-specific	[94]
2018	3D printing complex lattice structures for permeable liver phantom fabrication	Liver	[95]
2018	3D printing in personalized drug delivery	Non-specific	[96]
2018	3D Printing of PDMS Improves Its Mechanical and Cell Adhesion Properties	Breast	[97]
2018	3D-printed bioceramic scaffolds: From bone tissue engineering to tumor therapy	Bone	[98]
2018	3D-printed scaffolds with bioactive elements-induced photothermal effect for bone tumor therapy	Bone	[99]
2018	A bifunctional scaffold with CuFeSe 2 nanocrystals for tumor therapy and bone reconstruction	Bone	[100]
2018	An effective thermal therapy against cancer using an E-jet 3D-printing method to prepare implantable magnetocaloric mats	Non-specific	[101]
2018	Biomimetic 3D-printed custom-made prosthesis for anterior column reconstruction in the thoracolumbar spine: a tailored option following en bloc resection for spinal tumors: Preliminary results on a case-series of 13 patients	Spinal	[102]
2018	Bioprintable alginate/gelatin hydrogel 3D in vitro model systems induce cell spheroid formation	Non-specific	[103]
2018	Bioprinting of glioma stem cells improves their endotheliogenic potential	Brain	[104]
2018	Computer-aided designed, three dimensional-printed hemipelvic prosthesis for peri-acetabular malignant bone tumour	Bone	[105]

2018	Contribution of 3D printing to mandibular reconstruction after cancer	Head & Neck	[106]
2018	Current Use of Three-dimensional Model Technology in Urology: A Road Map for Personalised Surgical Planning	Prostate Kidney	[107]
2018	Design of spherically structured 3D in vitro tumor models -Advances and prospects	Non-specific	[108]
2018	Electrospinning: An enabling nanotechnology platform for drug delivery and regenerative medicine	Non-specific	[109]
2018	Emerging In Vitro 3D Tumour Models in Nanoparticle-Based Gene and Drug Therapy	Non-specific	[110]
2018	Emerging tumor spheroids technologies for 3D in vitro cancer modeling	Non-specific	[111]
2018	Engineering 3D approaches to model the dynamic microenvironments of cancer bone metastasis	Bone	[112]
2018	Halfway between 2D and animal models: Are 3D cultures the ideal tool to study cancer-microenvironment interactions?	Non-specific	[113]
2018	Implantation of a 3D-printed titanium sternum in a patient with a sternal tumor	Sternal	[114]
2018	In Vitro Study of Colon Cancer Cell Migration Using E-Jet 3D Printed Cell Culture Platforms	Colorectal	[115]
2018	Integration of 3D printing with dosage forms: A new perspective for modern healthcare	Non-specific	[116]
2018	Modelling glioma invasion using 3D bioprinting and scaffold-free 3D culture	Brain	[117]
2018	MR-CBCT image-guided system for radiotherapy of orthotopic rat prostate tumors	Prostate	[118]
2018	Patient-Specific Actual-Size Three-Dimensional Printed Models for Patient Education in Glioma Treatment: First Experiences	Brain	[119]
2018	Personalised 3D-printed model of a chest-wall chondrosarcoma to enhance patient understanding of complex cardiothoracic surgery	Chondrosarcom a	[120]
2018	Proof-of-Concept Study of 3-D–Printed Mold–Guided Breast-Conserving Surgery in Breast Cancer Patients	Breast	[121]
2018	Rapid 3D bioprinting of decellularized extracellular matrix with regionally varied mechanical properties and biomimetic microarchitecture	Liver	[122]
2018	Screening of additive manufactured scaffolds designs for triple negative breast cancer 3D cell culture and stem-like expansion	Breast	[123]
2018	Three-dimensional-printed individual template-guided 125I seed implantation for the cervical lymph node metastasis: A dosimetric and security study	Cervical lymph node	[124]
2018	Tumor-like lung cancer model based on 3D bioprinting	Lung	[125]
2018	Using 3D printing techniques to create an anthropomorphic thorax phantom for medical imaging purposes	Lung	[126]
2018	Using Three-Dimensional Printing to Create Individualized Cranial Nerve Models for Skull Base Tumor Surgery	Brain	[127]
2018	Virtual reality-assisted localization and three-dimensional printing-enhanced multidisciplinary decision to treat radiologically occult superficial endobronchial lung cancer	Lung	[128]
2018	Workload implications for clinic workflow with implementation of three-dimensional printed customized bolus for radiation therapy: A pilot study	Non-specific	[129]
2019	3D bioprinted glioma cell-laden scaffolds enriching glioma stem cells via epithelial–mesenchymal transition	Brain	[130]
2019	E-Jet 3D-Printed Scaffolds as Sustained Multi-Drug Delivery Vehicles in Breast Cancer Therapy. Pharmaceutical research	Breast	[131]
2019	Drop-on-powder 3d printing of tablets with an anti-cancer drug, 5-fluorouracil	Non-specific	[132]

2019	Novel design and development of a 3D-printed conformal superficial brachytherapy device for the treatment of non-melanoma skin cancer and keloids	Skin	[133]
2019	A Drug-Eluting 3D-Printed Mesh (GlioMesh) for Management of Glioblastoma. Advanced Therapeutics	Brain	[134]
2019	3D Bioprinted In Vitro Metastatic Models via Reconstruction of Tumor Microenvironments	Non-specific	[135]
2019	3D in vitro cancerous tumor models: Using 3D printers	Non-specific	[136]
2019	3D printing of hydrogel scaffolds for future application in photothermal therapy of breast cancer and tissue repair	Breast	[137]
2019	3D Printing of Poloxamer 407 Nanogel Discs and Their Applications in Adjuvant Ovarian Cancer Therapy	Gynaecological	[138]
2019	3D-Bioprinted Mini-Brain: A Glioblastoma Model to Study Cellular Interactions and Therapeutics	Brain	[139]
2019	3D-printed breast phantom for multi-purpose and multi-modality imaging	Breast	[140]
2019	3D-printed prosthesis replacement for limb salvage after radical resection of an ameloblastoma in the tibia with 1 year of follow up: A case report	Head & Neck	[141]
2019	A 3D bioprinted hydrogel mesh loaded with all-trans retinoic acid for treatment of glioblastoma	Brain	[142]
2019	Anticancer drug discovery using multicellular tumor spheroid models	Non-specific	[143]
2019	Bioengineering-inspired three-dimensional culture systems: Organoids to create tumor microenvironment	Non-specific	[144]
2019	Bioprinting a novel glioblastoma tumor model using a fibrin-based bioink for drug screening	Brain	[145]
2019	Combined Application of Modified Three-Dimensional Printed Anatomic Templates and Customized Cutting Blocks in Pelvic Reconstruction After Pelvic Tumor Resection	Pelvic	[146]
2019	Creating customized oral stents for head and neck radiotherapy using 3D scanning and printing	Head & Neck	[147]
2019	Current and Emerging 3D Models to Study Breast Cancer	Breast	[148]
2019	Decision-making based on 3D printed models in laparoscopic liver resections with intraoperative ultrasound: a prospective observational study	Liver	[149]
2019	Development and assessment of 3D-printed individual applicators in gynaecological MRI-guided brachytherapy	Gynaecological	[150]
2019	Digital applicator by 3D printing in contact brachytherapy	Non-specific	[151]
2019	Evaluation of mouthpiece fixation devices for head and neck radiotherapy patients fabricated in PolyJet photopolymer by a 3D printer	Head & Neck	[152]
2019	Exploring cancer cell behavior in vitro in three-dimensional multicellular bioprintable collagen-based hydrogels	Non-specific	[153]
2019	Impact of Three-dimensional Printing in Urology: State of the Art and Future Perspectives. A Systematic Review by ESUT-YAUWP Group	Non-specific	[154]
2019	Individualized 3D-printed templates for high-dose-rate interstitial multicathether brachytherapy in patients with breast cancer	Breast	[155]
2019	Laser-based 3D bioprinting for spatial and size control of tumor spheroids and embryoid bodies	Non-specific	[156]
2019	Modeling Tumor Phenotypes In Vitro with Three-Dimensional Bioprinting	Non-specific	[157]
2019	Personalized 3D-Printed Model for Informed Consent for Stage I Lung Cancer: A Randomized Pilot Trial	Lung	[158]
2019	Safety and efficacy of CT-guided radioactive iodine-125 seed implantation assisted by a 3D printing template for the treatment of thoracic malignancies	Thoracic	[159]

2019	Systematic review of the applications of three-dimensional printing in colorectal surgery	Colorectal	[160]
2019	The role of three-dimensional printing in the surgical management of breast cancer	Breast	[161]
2019	Three dimensional in vitro models of cancer: Bioprinting multilineage glioblastoma models	Brain	[162]
2019	Three-dimensional printing for laparoscopic partial nephrectomy in patients with renal tumors	Kidney	[163]
2019	Three-dimensional-printed vaginal applicators for electronic brachytherapy of endometrial cancers	Gynaecological	[164]
2019	Three-dimension-printed custom-made prosthetic reconstructions: from revision surgery to oncologic reconstructions	Non-specific	[165]
2019	Tumor Cells Develop Defined Cellular Phenotypes After 3D-Bioprinting in Different Bioinks	Skin	[166]
2020	3D bioprinted vascularized tumour for drug testing	Non-specfic	[167]
2020	A novel ex vivo trainer for robotic vesicourethral anastomosis	Bladder & Urethra	[168]
2020	3D Bioprinting of Tumor Models for Cancer Research	Non-specific	[169]
2020	3D printed biodegradable implants as an individualized drug delivery system for local chemotherapy of osteosarcoma	Bone	[170]
2020	3D printed core-shell hydrogel fiber scaffolds with NIR-triggered drug release for localized therapy of breast cancer	Breast	[171]
2020	3D printed in vitro tumor tissue model of colorectal cancer	Non-specific	[172]
2020	3D printed microfluidic devices for circulating tumor cells (CTCs) isolation	Breast<br>Prostate<br>Colorectal	[173]
2020	3D printed microneedles for anticancer therapy of skin tumours	Skin	[174]
2020	3D printing applications for the treatment of cancer	Non-specific	[175]
2020	3D printing of metal-organic framework nanosheets-structured scaffolds with tumor therapy and bone construction	Bone	[176]
2020	3D Printing, Augmented Reality, and Virtual Reality for the Assessment and Management of Kidney and Prostate Cancer: A Systematic Review	Kidney<br>Prostate	[177]
2020	3D-printed immunosensor arrays for cancer diagnostics	Non-specific	[178]
2020	A prospective parallel design study testing non-inferiority of customized oral stents made using 3D printing or manually fabricated methods	Head & Neck	[179]
2020	A review of 3D printed patient specific immobilisation devices in radiotherapy	Head & Neck	[180]
2020	A review on the application of 3d printing technology in radiotherapy for breast cancer	Breast	[181]
2020	Alginate-based hydrogels as drug delivery vehicles in cancer treatment and their applications in wound dressing and 3D bioprinting	Non-specific	[182]
2020	Analysis of principles inspiring design of three-dimensional-printed custom-made prostheses in two referral centres	Bone	[183]
2020	Application of 3D-Printed Craniocerebral Model in Simulated Surgery for Complex Intracranial Lesions	Brain	[184]
2020	Application of a 3D Bioprinted Hepatocellular Carcinoma Cell Model in Antitumor Drug Research	Liver	[185]
2020	Applications of 3D bioprinted-induced pluripotent stem cells in healthcare	Non-specific	[186]
2020	Bioprinted three-dimensional cell-laden hydrogels to evaluate adipocyte-breast cancer cell interactions	Breast	[187]

2020	Bioprinting of in vitro tumor models for personalized cancer treatment: a review	Non-specfic	[188]
2020	Bioprinting of patient-derivedin vitrointrahepatic cholangiocarcinoma tumor model: establishment, evaluation and anti-cancer drug testing	Bile duct	[189]
2020	Breast cancer models: Engineering the tumor microenvironment	Breast	[190]
2020	Clinical observation of 3D printing-guided three-dimensional brachytherapy for cervical cancer	Gynaecological	[191]
2020	Could 3D models of cancer enhance drug screening	Non-specific	[192]
2020	Cryogenic 3D printing of porous scaffolds for in situ delivery of 2D black phosphorus nanosheets, doxorubicin hydrochloride and osteogenic peptide for treating tumor resection-induced bone defects	Bone	[193]
2020	Current practice in preoperative virtual and physical simulation in neurosurgery	Brain	[194]
2020	Decision-making based on 3D printed models in laparoscopic liver resections with intraoperative ultrasound: a prospective observational study	Liver	[195]
2020	Design and Fabrication of Three-Dimensional Printed Scaffolds for Cancer Precision Medicine	Non-specfic	[196]
2020	Design, modeling and 3D printing of a personalized cervix tissue implant with protein release function	Gynaecological	[197]
2020	Development of a bioprinting approach for automated manufacturing of multi-cell type biocomposite TRACER strips using contact capillary-wicking	Non-specific	[198]
2020	E-jet 3D printed drug delivery implants to inhibit growth and metastasis of orthotopic breast cancer	Breast	[199]
2020	Engineering a Novel 3D Printed Vascularized Tissue Model for Investigating Breast Cancer Metastasis to Bone	Breast	[200]
2020	Engineering bioprintable alginate/gelatin composite hydrogels with tunable mechanical and cell adhesive properties to modulate tumor spheroid growth kinetics	Breast	[201]
2020	Engineering Three-Dimensional Tumor Models to Study Glioma Cancer Stem Cells and Tumor Microenvironment	Brain	[202]
2020	Geometrically Structured Microtumors in 3D Hydrogel Matrices	Skin	[203]
2020	Immersion bioprinting of tumor organoids in multi-well plates for increasing chemotherapy screening throughput	Non-specfic	[204]
2020	Implementation of the three-dimensional printing technology in treatment of bone tumours: a case series	Bone	[205]
2020	Improving Bioprinted Volumetric Tumor Microenvironments In Vitro	Non-specific	[206]
2020	Investigating lymphangiogenesis in a sacrificially bioprinted volumetric model of breast tumor tissue	Breast	[207]
2020	Laser-assisted 3D bioprinting of exocrine pancreas spheroid models for cancer initiation study	Pancreatic	[208]
2020	Methodology of custom design and manufacturing of 3D external breast prostheses	Breast	[209]
2020	Multi-colour extrusion fused deposition modelling: a low-cost 3D printing method for anatomical prostate cancer models	Prostate	[210]
2020	Multimaterial three-dimensional printing in brachytherapy: Prototyping teaching tools for interstitial and intracavitary procedures in cervical cancers	Gynaecological	[211]
2020	New Surgical Approaches in the Treatment of Non–Small Cell Lung Cancer	Lung	[212]

2020	Novel 3D printed device with integrated macroscale magnetic field triggerable anti-cancer drug delivery system	Non-specfic	[213]
2020	Organotypic cancer tissue models for drug screening: 3D constructs, bioprinting and microfluidic chips	Non-specfic	[214]
2020	Patient-Derived In Vitro Models for Drug Discovery in Colorectal Carcinoma	Non-specific	[215]
2020	Recent Advances in 3D Bioprinted Tumor Microenvironment	Non-specfic	[216]
2020	Recent advances on utilization of bioprinting for tumor modeling	Non-specfic	[217]
2020	The construction of in vitro tumor models based on 3D bioprinting	Non-specfic	[218]
2020	The role of a drug-loaded poly (lactic co-glycolic acid) (PLGA) copolymer stent in the treatment of ovarian cancer	Gynaecological	[219]
2020	The Use of 3D Printed Microporous-Strut Polycaprolactone Scaffolds for Targeted Local Delivery of Chemotherapeutic Agent for Breast Cancer Application	Breast	[220]
2020	Thermal Bioprinting Causes Ample Alterations of Expression of LUCAT1, IL6, CCL26, and NRN1L Genes and Massive Phosphorylation of Critical Oncogenic Drug Resistance Pathways in Breast Cancer Cells	Breast	[221]
2020	Three dimensional in vitro models of cancer: Bioprinting multilineage glioblastoma models	Brain	[222]
2020	Three-dimensional bioprinted glioblastoma microenvironments model cellular dependencies and immune interactions.	Brain	[223]
2020	Three-dimensional printed silicone bite blocks for radiotherapy of head and neck cancer-a preliminary study	Head & Neck	[224]
2020	Three-Dimensional Printing for Chest Wall Reconstruction in Thoracic Surgery: Building on Experience	Lung	[225]
2020	Three-dimensional reconstruction/personalized three-dimensional printed model for thoracoscopic anatomical partial-lobectomy in stage i lung cancer: A retrospective study	Lung	[226]
2020	Use of 3-Dimensional Printing Technology in Complex Spine Surgeries.	Spinal	[227]
2020	Use of personalized 3D printed kidney models for partial nephrectomy	Kidney	[228]
2020	Usefulness of a 3D-Printed Thyroid Cancer Phantom for Clinician to Patient Communication	Thyroid	[229]
2021	3D Bioprinted cancer models: Revolutionizing personalized cancer therapy	Non-specific	[230]
2021	3D bioprinting for in vitro models of oral cancer: Toward development and validation	Head & Neck	[231]
2021	3D bioprinting of engineered breast cancer constructs for personalized and targeted cancer therapy	Breast	[232]
2021	3D Bioprinting of Model Tissues That Mimic the Tumor Microenvironment	Breast	[233]
2021	3D Printing in Breast Reconstruction: From Bench to Bed	Breast	[234]
2021	3D printing novel in vitro cancer cell culture model systems for lung cancer stem cell study	Lung	[235]
2021	A 3D Bioprinted Material That Recapitulates the Perivascular Bone Marrow Structure for Sustained Hematopoietic and Cancer Models	Breast	[236]
2021	Acceptability of 3D-printed breast models and their impact on the decisional conflict of breast cancer patients: A feasibility study	Breast	[237]
2021	Advanced Spheroid, Tumouroid and 3D Bioprinted In-Vitro Models of Adult and Paediatric Glioblastoma	Brain	[238]

2021	Advances in 3D bioprinting for the biofabrication of tumor models	Non-specific	[239]
2021	Alginate-gelatin-Matrigel hydrogels enable the development and multigenerational passaging of patient-derived 3D bioprinted cancer spheroid models	Non-specific	[240]
2021	Artificial Tumor Microenvironments in Neuroblastoma	Non-specific	[241]
2021	Bioprinting and Differentiation of Adipose-Derived Stromal Cell Spheroids for a 3D Breast Cancer-Adipose Tissue Model	Breast	[242]
2021	Bioprinting on Live Tissue for Investigating Cancer Cell Dynamics	Breast	[243]
2021	Cancer Cell Direct Bioprinting: A Focused Review	Non-specific	[244]
2021	Comprehensive review of 3D printing use in medicine: Comparison with practical applications in urology	Kidney	[245]
2021	Cytotoxic and chemosensitizing effects of glycoalkaloidic extract on 2D and 3D models using RT4 and patient derived xenografts bladder cancer cells	Bladder	[246]
2021	Detecting cancer metastasis and accompanying protein biomarkers at single cell levels using a 3D-printed microfluidic immunoarray	Head & Neck	[247]
2021	Development of A 3D-Printed Navigational Template for Establishing Rabbit VX2 Lung Cancer Model	Lung	[248]
2021	Development of a Tongue Immobilization Device Using a 3D Printer for Intensity Modulated Radiation Therapy of Nasopharyngeal Cancer Patients	Head & Neck	[249]
2021	Difficult airways: a 3D printing study with virtual fibreoptic endoscopy	Head & Neck	[250]
2021	Dosimetric comparison of computed tomography-guided iodine-125 seed implantation assisted with and without three-dimensional printing non-coplanar template in locally recurrent rectal cancer: a propensity score matching study	Colorectal	[251]
2021	Evolution of Metastasis Study Models toward Metastasis-On-A-Chip: The Ultimate Model?	Non-specific	[252]
2021	Introduction to bioprinting of in vitro cancer models	Non-specific	[253]
2021	Modeling the mechanobiology of cancer cell migration using 3D biomimetic hydrogels	Non-specific	[254]
2021	Multi-institutional validation of a perfused robot-assisted partial nephrectomy procedural simulation platform utilizing clinically relevant objective metrics of simulators (CROMS)	Kidney	[255]
2021	Patient-specific desktop 3D-printed guides for pelvic tumour resection surgery: a precision study on cadavers	Pelvic	[256]
2021	Patient-Specific Quality Assurance Using a 3D-Printed Chest Phantom for Intraoperative Radiotherapy in Breast Cancer	Breast	[257]
2021	Printing the Pathway Forward in Bone Metastatic Cancer Research: Applications of 3D Engineered Models and Bioprinted Scaffolds to Recapitulate the Bone-Tumor Niche	Breast	[258]
2021	Programmable shape transformation of 3D printed magnetic hydrogel composite for hyperthermia cancer therapy	Skin	[259]
2021	Re-interpreting mesenteric vascular anatomy on 3D virtual and/or physical models: positioning the middle colic artery bifurcation and its relevance to surgeons operating colon cancer	Colorectal	[260]
2021	Studying Tumor Angiogenesis and Cancer Invasion in a Three-Dimensional Vascularized Breast Cancer Micro-Environment	Breast	[261]
2021	The accuracy and dosimetric analysis of 3D-printing non-coplanar template-assisted iodine-125 seed implantation for recurrent chest wall cancer	Chest wall	[262]

2021	The Accuracy of Individualized 3D-Printing Template-Assisted I125 Radioactive Seed Implantation for Recurrent/Metastatic Head and Neck Cancer	Head & Neck	[263]
2021	The dosimetry evaluation of 3D printing non-coplanar template-assisted CT-guided 125I seed stereotactic ablation brachytherapy for pelvic recurrent rectal cancer after external beam radiotherapy	Colorectal	[264]
2021	Three-dimensional computed tomography angiography and bronchography combined with three-dimensional printing for thoracoscopic pulmonary segmentectomy in stage IA non-small cell lung cancer	Lung	[265]
2021	Total Talar Replacement Using a Novel 3D-Printed Prosthesis for Recurrent Giant Cell Tumour of the Talus	Bone	[266]

Table S2. Clinical trials related to cancer utilising 3D printing technologies by country.

Trial ID	Title	Primary sponsor	Country	Year
NCT04176900	3D Printed Rigid Bolus Versus Silicone Bolus for Treatment of Tumors Involving the Skin: A Comparative Study	Nova Scotia Cancer Centre	CA	2020
NCT04098146	Registry to Collect Data on Patients Undergoing Segmental Mandibular Defect Reconstruction Following Oral Squamous Cell Carcinoma Resection	AO Clinical Investigation and Documentation	CH	2020
NCT03738488	3D-biomodels for Surgical Planning in Patients With Renal Cancer and Vascular Involvement	Fundación Pública Andaluza para la gestión de la Investigación en Sevilla	ES	2018
NCT03943771	Effect on Pre-operative Anxiety of a Personalized Three-dimensional Kidney Model Prior to Nephron-sparing Surgery for Renal Tumor.	University Hospital, Bordeaux	FR	2019
KCT0002375	The application of 3D printing surgical guide based on MRI and 3D scanner for breast cancer precision surgery to breast conserving surgery and verification of the usefulness of volume navigation ultrasound	Asan Medical Center	KR	2017
KCT0002274	MRI and volume navigation ultrasound are used to track the size and location of tumors according to treatment and postural changes in patients with breast cancer who received neoadjuvant chemotherapy	Asan Medical Center	KR	2017
KCT0002272	Availability of 3D printing guide during breast conserving surgery with breast cancer patients who had neoadjuvant treatment	Asan Medical Center	KR	2017
KCT0003043	Development of automated tumor tracking system using artificial intelligence in breast cancer patients and improvement of breast conservation through 3D printing surgery guide and validation of volume navigation ultrasound	Asan Medical Center	KR	2018
KCT0004469	A randomized, prospective, multicenter study to validate the usefulness of 3D-printing surgical guides for breast-conserving surgery in breast cancer patients treated with neoadjuvant chemotherapy	Asan Medical Center	KR	2019

KCT0004705	An Prospective analysis of clinical effectiveness for 3D printed, customized surgical skin cancer resection guide	Asan Medical Center	KR	2020
NCT03744624	Application of 3D Printing in Laparoscopic Surgery of Liver Tumors	Jagiellonian University	PL	2018
NCT02901782	The Application of Personalized Titanium Plate in the Bone Tumors Around the Knee	Guangzhou General Hospital of Guangzhou Military Command丨Medical Center of Assessment, Prevention and Treatment of Bone & Joint Diseases, Guangdong, China丨The First Affiliated Hospital of Guangzhou Medical University	PRC	2013
ChiCTR-INR-16009479	Application of 3D printing technique in small pulmonary nodule localization	Shanghai Pulmonary Hospital	PRC	2016
ChiCTR-INR-16009449	Development and application of 3D printing technology in oral mould system	Ninth Peoples' Hospital, Medical school, Jiaotong University	PRC	2016
ChiCTR-INR-16010294	The multicenter, randomized, open and positive control study to realize the individualized limb-salvage reconstruction of the pelvic tumors based on 3D printing technology	Ninth People's Hospital, Shanghai Jiaotong University School of Medicine	PRC	2016
NCT03057223	Three-Dimensional Printing of Patient-Specific Titanium Plates in Jaw Surgery: A Pilot Study	The University of Hong Kong丨Chinese University of Hong Kong	PRC	2016
NCT03153332	Value of 3D Printing for Comprehension of Liver Surgical Anatomy	Guangzhou Women and Children's Medical Center	PRC	2017
NCT03348293	Safety Study of 3D Printing Personalized Biodegradable Implant for Breast Reconstruction	Xijing Hospital	PRC	2017
NCT03325907	Transthoracic Needle Biopsy Using 3D Printed Navigational Template: A Phase I Feasibility Trial	Shanghai Pulmonary Hospital, Shanghai, China	PRC	2017
ChiCTR1800017161	Application of three-dimensional reconstruction and 3D printing of superior mesenteric vessels in laparoscopic CME surgery for right colon cancer	Wuxi Second People's Hospital	PRC	2018
NCT03599895	Application of 3D Printing Technique in Radical Gastrectomy With Leonardo da Vinci Robot	First Affiliated Hospital Xi'an Jiaotong University	PRC	2018
ChiCTR1800014561	Combined application of a modified 3D-printed anatomic template and customized cutting block in pelvic reconstruction after zones II and III borderline pelvic tumor resection	Chongqing Traditional Chinese Medicine Hospital	PRC	2018
ChiCTR1800015820	The randomized, open and positive control study for imaging surgical planning of the pelvic tumors based on 3D printing technology	Ninth People's Hospital, Shanghai Jiaotong University School of Medicine	PRC	2018
NCT04056923	3D Printing for Nodule Localization	Wen-zhao ZHONG丨Zhuhai Seine Technology Co., Ltd, Zhuhai city, China丨GuangdongPPH	PRC	2018

NCT03890926	The Physical Dosimetry Study and Preliminary Clinical Results of 3D-printing Non Co-planar Template Assisted With CT-guidance for Iodine-125 Seed Brachytherapy in Pelvic Recurrent Rectum Carcinoma After Surgery and External Beam Radiotherapy	Peking University Third Hospital	PRC	2019
NCT03964064	I125 Seed Implantation vs Stereotactic Radiotherapy for Pancreatic Cancer	Peking University Third Hospital	PRC	2019
NCT04071418	I-125 Seeds Implantation in the Treatment of Recurrent Lung Cancer After Radiotherapy	Peking University Third Hospital	PRC	2019
NCT03890575	Airway Stent Modified With 3D Printing for Malignant Stricture Involving Carina and Distal Bronchi	Ruijin Hospital	PRC	2019
NCT04127435	High Dose Rate Interstitial Brachytherapy With Three Dimensional Printing Template for Recurrent Gynecologic Tumors	Peking University Third Hospital	PRC	2019
NCT04077710	RISI in the Treatment of Recurrent Chest Wall Malignancies After EBRT	Peking University Third Hospital	PRC	2019
NCT03963726	Clinical Efficacy of Stereotactic Radiotherapy and Microwave Ablation for Liver Metastases From Colorectal Cancer.	Peking University Third Hospital丨The fifth medical center of PLA general hospital丨Tengzhou Central People's Hospital丨Guangxi Ruikang Hospital丨Hunan Aerospace Hospital丨Beijing Ditan Hospital	PRC	2019
NCT03882866	Iodine-125 Seed Implantation Therapy for Locally Advanced Pancreatic Cancer	Ruijin Hospital丨Hebei General Hospital丨Zhongda Hospital丨Anhui Provincial Hospital丨The Second Hospital of Shandong University丨Sun Yat-sen University丨Chinese PLA General Hospital丨Huadong Hospital丨Tengzhou Central People's Hospital	PRC	2019
ChiCTR2000038145	A non randomized controlled study of 3D printing artificial vertebral body in the treatment of spinal tumors	Zhongnan Hospital Of Wuhan University	PRC	2020
ChiCTR2000039996	Clinical application of 3D printing technology in breast cancer: breast-conserving margin evaluation and new adjuvant therapy withdrawal model establishment	Shenzhen Second People's Hospital	PRC	2020
ChiCTR2000035858	Development and clinical application of 3D printing universal iliac wing prosthesis	Shanghai Sixth People's Hospital	PRC	2020
NCT04552054	Mixed Reality Technique Combined With 3D Printing Navigational Template for Localizing Pulmonary Nodules	Guangdong Provincial People's Hospital	PRC	2020
NCT04266327	RISI in the Treatment of Recurrent Metastatic SCC of Thoracic Inlet Lymph Nodes	Peking University Third Hospital	PRC	2020
ChiCTR2000039527	The safety and efficacy of navigation system combined with 3D-printing template assisted CT-guided radioactive seed implantation in the treatment of spinal tumors	Peking University Third Hospital	PRC	2020

ChiCTR2000034564	Using 3D reconstruction / 3D printing model to assist pathological sampling of multifocal nodules: a prospective cohort study	National Cancer Center/National Clinical Research Center for Cancer/Cancer Hospital, Chinese Academy of Medical Sciences and Peking Union Medical College	PRC	2020
NCT04635865	3D-Printed Patient-Specific Surgical Plates Versus Conventional Surgical Plates in Jaw Reconstruction	The University of Hong Kong	PRC	2021
NCT04710589	CTV Exploration of 3D-PT Assisted CT-guided I-125 Seeds Implantation for Recurrent Rectal Cancer	Peking University Third Hospital	PRC	2021
NCT03466957	Individual Approach in Gynecological Cancer Brachytherapy	Institute of Oncology Ljubljana	SI	2016
NCT02952261	Application of 3D Printing Technique in Small Pulmonary Nodule Localization	Chang Chen	TW	2016
NCT04929197	Evaluation of the Feasibility of Developing Personalized Breast Cancer Radiotherapy Assistive Device With 3D Printing	Taipei Medical University Hospital	TW	2021
ISRCTN75603704	The "R-3D-2" pilot study - the impact of rehearsal strategies prior rectal cancer surgery, using patient individualised 3D models	University of Leeds	UK	2015
NCT02930915	Acquisition of 3D Facial Geometry of Patients' Scheduled for Radiotherapy Treatment	University of East Anglia丨Clinical Research and Trials Unit (Norfolk & Norwich University Hospital, UK)	UK	2016
NCT02550210	A Study to Evaluate the Accuracy of a Breast Cancer Locator (BCL) in Patients With Palpable Cancers	Dartmouth-Hitchcock Medical Center	USA	2015
NCT03573661	A Pilot Multi-Institutional Study to Evaluate the Accuracy of a Breast Cancer Locator in Patients With Palpable Cancers	Dartmouth-Hitchcock Medical Center	USA	2018

Abbreviations: CA—Canada; CH—Switzerland; ES—Spain; FR—France; KR—South Korea; PL—Poland; PRC—People'sRepublic of China; SI—Slovenia; TW—Taiwan; UK—United Kingdom; USA—United States of America.

Table S3. Patent of 3D printing technologies for cancer applications according to year of publication.

Publication Number	Title	Year	Country
WO/2015/013716	Patient-Specific Temporary Implants for Accurately Guiding Local Means of Tumor Control along Patient-Specific Internal Channels to Treat Cancers	2015	USA
106182774	Method for Printing Liver Cancer Model by Three-Dimensional (3D) Printing Technology and Liver Cancer Model	2016	China
106109245	Anti-Cancer Drug Prepared by Using 3D Printing Technology, and Method	2016	China
201610390333.9	Cervical Cancer Entity Model Making Method	2016	China
105997313	3D Printed Breast External Prosthesis and Manufacturing Method	2016	China
106110515	3D Conformal Chest Wall Radiotherapy Tissue Compensation Technology after Breast Cancer Surgery	2016	China
106228884	Anthropomorphic Phantom for Individualization Radiotherapy Dosage Verification	2016	China
106236185	3D Printed Fibula Cutting Device and Manufacturing and Use Method Thereof	2016	China
105944141	Cervix Bionic Matter Used for Transplanting and Preparation Method Thereof	2016	China

105892959	Method for Customizing Personalized Silica Gel Prosthesis Mold on Basis Of MRI (Magnetic Resonance Imaging) Image Data Of Breasts	2016	China
20160271379	Patient-Specific Temporary Implants for Accurately Guiding Local Means of Tumor Control along Patient-Specific Internal Channels to Treat Cancer	2016	USA
107007295	Early Diagnosis Device of Micro-Nodular Lung Cancer	2017	China
WO/2017/200298	Remote Afterloading Brachytherapy Apparatus Using Liquid Radioactive Isotope	2017	South Korea
106964076	Radiotherapy Body Position Fixing Device Manufacturing Method and System Based on Three-Dimensional Scanning	2017	China
1017838810000*	Remote Back-Mounted Radiation Proximity Therapy Device Using Liquid Radioactive Isotope	2017	South Korea
109432612	Positioning Device For Breast Cancer Prone Treatment Based On 3D Printing And Preparation Method Thereof	2018	China
108670410	Lung Cancer Surgery Auxiliary Device	2018	China
108888876	3D-Printing-Based Stray Radiation Protector Out Of Radiotherapy Radiation Field, And Manufacturing Method Thereof	2018	China
201810020385.6	Method For Preparing Cutting Guide Plate Used For Determining Size Of Substitutional Flap On Surface Of Soft Tissue By Utilizing Digital Space Reconstruction And 3D Printing	2018	China
108339181	Method For Preparing Trachea Cannula By Utilizing Digital Space Reconstruction And 3D Printing Technology	2018	China
108670362	Methods For Preparing Cutting Guide Plates And Determining Cut Soft Tissue Substituting Flap Volume By Using Digital Space Reconstruction And 3D Printing Technology	2018	China
108888875	Whole Body Radiotherapy Tissue Compensation Device And Manufacturing Method Thereof	2018	China
201811041481.5	Body Surface Positioning Guide Plate For Guiding Radiation Particles In Short-Distance Cancer Treatment And Manufacturing Method Thereof	2018	China
108831269	Quality Control Detection Training Phantom For Bladder Capacity Measuring Instrument And Manufacturing Method Of Quality Control Detection Training Phantom	2018	China
109925614	Radiotherapy Headrest Based On 3D Printing And Radiotherapy Headrest Manufacturing Method And Manufacturing Device	2019	China
209270659	Breast Cancer Prone Treatment Positioning Device Based On 3D Printing	2019	China
109717999	3D Printing Improved Type Parastomal Hernia Abdominal Belt And 3D Printing Device Thereof	2019	China
209847516	3D Printed Improved Para-Stoma Hernia Bellyband And 3D Printing Device Thereof	2019	China
WO/2019/232114	Injectable Thermoresponsive Hydrogels As A Combinatory Modality For Controlled Drug Delivery, Biomaterial Implant And 3d Printing Bioink	2019	USA
209019793	3D Printing-Based Radiotherapy Radiation Field Stray Radiation Protection Device	2019	China
109876305	3D Printing Method For Customizing Silica Gel Tissue Compensation	2019	China
201910513761.X	Medical Curved Needle Puncture Method Based On 3D Printing Navigation Template	2019	China
109662806	Rib Locking Type Scapula Prosthesis Based On 3D Printing	2019	China
WO/2019/195394	Drug Screening Platform Using Biomaterial Scaffolds	2019	USA
WO/2019/219311	Analyzer Device For In Vitro Diagnostics	2019	Germany
209596423	Positioning And Guiding Device For Cervical Cancer Close-Range Treatment Minimally Invasive Surgery	2019	China
110314288	In-Cavity Positioning Guide Plate For Guiding Radioactive Particle In Brachytherapy Of Cancer And Making Method	2019	China

109363820	Auxiliary Making Device For User-Friendly Bone Clamping Plate And Bone Clamping Plate Making Method	2019	China
110302432	Method For Preparing Full-Thickness Skin Tissue Engineering Scaffold With Gradient Pore Structure	2019	China
11529919	Novel Gynecological Tumor 3d Printing Implantation Auxiliary Device	2020	China
111467692	Prone Position Positioning Device For Breast Cancer Radiotherapy, Shaped Through 3dprinting	2020	China
111298193	Stent, Preparation Method Thereof And Application Thereof In Breast Cancer Treatment And Bone Repair<br>Cn - 19.06.2020	2020	China
210190627	Ovel 3d Printing Head, Neck And Elbow Arm Bracket	2020	China
210136121	3d Printing Specimen Sampling Box For Manufacturing Large Prostate Cancer Pathological Sections	2020	China
20210205459	Injectable Thermoresponsive Hydrogels As A Combinatory Modality For Controlled Drug Delivery, Biomaterial Implant And 3d Printing Bioink	2021	USA
WO/2021/123344	Injectable Thermoresponsive Hydrogels As A Combinatory Modality For Controlled Drug Delivery, Biomaterial Implant And 3d Printing Bioink	2021	Ireland
2021047813	Drug Screening Platform Using Biomaterial Scaffolds	2021	USA
112336684	Preparation Method Of 5-Fluorouracil/Ultra-High Molecular Weight Polyethylene Anti-Tumor Implant Stent<br>Cn - 09.02.2021	2021	China
212416696	Prostrate Position Positioning Device For Breast Cancer Radiotherapy Formed Through 3dprinting<br>Cn - 29.01.2021	2021	China
112220544	Pedicle Screw Rod System And Manufacturing Method Thereof	2021	China
212331859	3d Printing Device Based On Tumor Preoperative Reconstruction Model	2021	China

References

1. Cohen, A., et al., *Mandibular reconstruction using stereolithographic 3-dimensional printing modeling technology.* Oral Surgery, Oral Medicine, Oral Pathology, Oral Radiology, and Endodontology, 2009. **108**(5): p. 661-666.
2. Xu, F., et al., *A three-dimensional in vitro ovarian cancer coculture model using a high-throughput cell patterning platform.* Biotechnology Journal, 2011. **6**(2): p. 204-212.
3. Tam, M.D., et al., *3-D printout of a DICOM file to aid surgical planning in a 6 year old patient with a large scapular osteochondroma complicating congenital diaphyseal aclasia.* Journal of Radiology Case Reports, 2012. **6**(1): p. 31-37.
4. Lee, B.K., et al., *Fabrication of drug-loaded polymer microparticles with arbitrary geometries using a piezoelectric inkjet printing system.* International journal of pharmaceutics, 2012. **427**(2): p. 305-310.
5. Chen, M., et al., *Fabrication and characterization of a rapid prototyped tissue engineering scaffold with embedded multicomponent matrix for controlled drug release.* International journal of nanomedicine, 2012. **7**: p. 4285.
6. Spottiswoode, B., et al., *Preoperative Three-Dimensional Model Creation of Magnetic Resonance Brain Images as a Tool to Assist Neurosurgical Planning.* Stereotactic and Functional Neurosurgery, 2013. **91**(3): p. 162-169.
7. Chae, M.P., et al., *3D volumetric analysis for planning breast reconstructive surgery.* Breast Cancer Research and Treatment, 2014. **146**(2): p. 457-460.
8. Watson, R.A., *A Low-Cost Surgical Application of Additive Fabrication.* Journal of Surgical Education, 2014. **71**(1): p. 14-17.
9. Zein, N.N., et al., *Three-dimensional print of a liver for preoperative planning in living donor liver transplantation.* Liver Transplantation, 2013. **19**(12): p. 1304-1310.
10. Zhang, J., et al., *3D-printed magnetic Fe 3 O 4/MBG/PCL composite scaffolds with multifunctionality of bone regeneration, local anticancer drug delivery and hyperthermia.* Journal of Materials Chemistry B, 2014. **2**(43): p. 7583-7595.
11. Zhao, Y., et al., *Three-dimensional printing of Hela cells for cervical tumor model in vitro.* Biofabrication, 2014. **6**(3).
12. Bersini, S. and M. Moretti, *3D functional and perfusable microvascular networks for organotypic microfluidic models.* Journal of Materials Science: Materials in Medicine, 2015. **26**(5).
13. Zhao, X., et al. *Anti-Cancer Drug Screening Based on a Adipose-Derived Stem Cell/Hepatocyte 3D Printing Technique*. 2015. Journal of Stem Cell Research & Therapy.
14. Lee, V.K., et al. *Generation of 3-D glioblastoma-vascular niche using 3-D bioprinting*. 2015. Institute of Electrical and Electronics Engineers Inc.
15. Lu, Y., et al., *Microstereolithography and characterization of poly (propylene fumarate)-based drug-loaded microneedle arrays.* Biofabrication, 2015. **7**(4): p. 045001.
16. Uddin, M.J., et al., *Inkjet printing of transdermal microneedles for the delivery of anticancer agents.* International journal of pharmaceutics, 2015. **494**(2): p. 593-602.
17. Agila, S. and J. Poornima. *Magnetically controlled nano-composite based 3D printed cell scaffolds as targeted drug delivery systems for cancer therapy*. in *2015 IEEE 15th International Conference on Nanotechnology (IEEE-NANO)*. 2015. IEEE.
18. Zachkani, P., et al., *A cylindrical magnetically-actuated drug delivery device proposed for minimally invasive treatment of prostate cancer.* RSC Advances, 2015. **5**(119): p. 98087-98096.
19. Lu, Y.Z., et al., *3D-printing microfludic device for breast cancer cell culture and pharmacological research.* Chinese Pharmaceutical Journal, 2015. **50**(24): p. 2124-2129.
20. Knowlton, S., et al., *Bioprinting for cancer research.* Trends in Biotechnology, 2015. **33**(9): p. 504-513.
21. Knoedler, M., et al., *Individualized physical 3-dimensional kidney tumor models constructed from 3-dimensional printers result in improved trainee anatomic understanding.* Urology, 2015. **85**(6): p. 1257-1262.
22. Mou, H., et al., *Non-small cell lung cancer 95D cells co-cultured with 3D-bioprinted scaffold to construct a lung cancer model in vitro.* Zhonghua zhong liu za zhi [Chinese journal of oncology], 2015. **37**(10): p. 736-740.
23. Kim, M.P., et al., *Three dimensional model for surgical planning in resection of thoracic tumors.* International Journal of Surgery Case Reports, 2015. **16**: p. 127-129.
24. Wang, Y., et al., *Three-dimensional printing technique assisted cognitive fusion in targeted prostate biopsy.* Asian Journal of Urology, 2015. **2**(4): p. 214-219.
25. Aranda, J.L., et al., *Tridimensional titanium-printed custom-made prosthesis for sternocostal reconstruction.* European Journal of Cardio-thoracic Surgery, 2015. **48**(4): p. e92-e94.
26. Dai, X., et al., *3D bioprinted glioma stem cells for brain tumor model and applications of drug susceptibility.* Biofabrication, 2016. **8**(4).
27. Javan, R., D. Herrin, and A. Tangestanipoor, *Understanding Spatially Complex Segmental and Branch Anatomy Using 3D Printing: Liver, Lung, Prostate, Coronary Arteries, and Circle of Willis.* Academic Radiology, 2016. **23**(9): p. 1183-1189.
28. Zhu, W., et al., *3D printed nanocomposite matrix for the study of breast cancer bone metastasis.* Nanomedicine: Nanotechnology, Biology, and Medicine, 2016. **12**(1): p. 69-79.

29. Jang, J., H.G. Yi, and D.W. Cho, *3D Printed Tissue Models: Present and Future.* ACS Biomaterials Science and Engineering, 2016. **2**(10): p. 1722-1731.
30. Ma, H., et al., *3D printing of biomaterials with mussel-inspired nanostructures for tumor therapy and tissue regeneration.* Biomaterials, 2016. **111**: p. 138-148.
31. Yi, H.G., et al., *A 3D-printed local drug delivery patch for pancreatic cancer growth suppression.* Journal of Controlled Release, 2016. **238**: p. 231-241.
32. Panagiotakopoulou, M., et al., *A Nanoprinted Model of Interstitial Cancer Migration Reveals a Link between Cell Deformability and Proliferation.* ACS Nano, 2016. **10**(7): p. 6437-6448.
33. Park, S.Y., et al., *A patient-specific polylactic acid bolus made by a 3D printer for breast cancer radiation therapy.* PLoS ONE, 2016. **11**(12).
34. Ozbolat, I.T., W. Peng, and V. Ozbolat, *Application areas of 3D bioprinting.* Drug Discovery Today, 2016. **21**(8): p. 1257-1271.
35. Wake, N., et al., *Application of anatomically accurate, patient-specific 3D printed models from MRI data in urological oncology.* Clinical Radiology, 2016. **71**(6): p. 610-614.
36. Zhu, W., et al., *3D printed nanocomposite matrix for the study of breast cancer bone metastasis.* Nanomedicine: Nanotechnology, Biology and Medicine, 2016. **12**(1): p. 69-79.
37. Lovitt, C.J., T.B. Shelper, and V.M. Avery, *Cancer drug discovery: recent innovative approaches to tumor modeling.* Expert Opinion on Drug Discovery, 2016. **11**(9): p. 885-894.
38. Canters, R.A., et al., *Clinical implementation of 3D printing in the construction of patient specific bolus for electron beam radiotherapy for non-melanoma skin cancer.* Radiotherapy and Oncology, 2016. **121**(1): p. 148-153.
39. Wang, H., et al., *CT guidance 125I seed implantation for pelvic recurrent rectal cancer assisted by 3D printing individual non-coplanar template.* National Medical Journal of China, 2016. **96**(47): p. 3782-3786.
40. Zhu, W., et al., *A 3D printed nano bone matrix for characterization of breast cancer cell and osteoblast interactions.* Nanotechnology, 2016. **27**(31): p. 315103.
41. Chen, X., et al., *Image-guided installation of 3D-printed patient-specific implant and its application in pelvic tumor resection and reconstruction surgery.* Computer Methods and Programs in Biomedicine, 2016. **125**: p. 66-78.
42. Lindegaard, J.C., et al., *Individualised 3D printed vaginal template for MRI guided brachytherapy in locally advanced cervical cancer.* Radiotherapy and Oncology, 2016. **118**(1): p. 173-175.
43. Wendler, J.J., et al., *Irreversible Electroporation of Prostate Cancer: Patient-Specific Pretreatment Simulation by Electric Field Measurement in a 3D Bioprinted Textured Prostate Cancer Model to Achieve Optimal Electroporation Parameters for Image-Guided Focal Ablation.* CardioVascular and Interventional Radiology, 2016. **39**(11): p. 1668-1671.
44. Bernhard, J.C., et al., *Personalized 3D printed model of kidney and tumor anatomy: a useful tool for patient education.* World Journal of Urology, 2016. **34**(3): p. 337-345.
45. Clark, M., B. Ghammraoui, and A. Badal. *Reproducing 2D breast mammography images with 3D printed phantoms*. 2016. SPIE.
46. Nath, S. and G.R. Devi, *Three-dimensional culture systems in cancer research: Focus on tumor spheroid model.* Pharmacology and Therapeutics, 2016. **163**: p. 94-108.
47. Jentzsch, T., et al., *Tumor resection at the pelvis using three-dimensional planning and patient-specific instruments: A case series.* World Journal of Surgical Oncology, 2016. **14**(1).
48. Albritton, J.L. and J.S. Miller, *3D bioprinting: Improving in vitro models of metastasis with heterogeneous tumor microenvironments.* DMM Disease Models and Mechanisms, 2017. **10**(1): p. 3-14.
49. Salmoria, G., et al., *3D printing of PCL/Fluorouracil tablets by selective laser sintering: Properties of implantable drug delivery for cartilage cancer treatment.* drugs. **4**: p. 6.
50. Salmoria, G., P. Klauss, and L. Kanis, *Laser Printing of PCL/Progesterone Tablets for Drug Delivery Applications in Hormone Cancer Therapy.* Lasers in Manufacturing and Materials Processing, 2017. **4**(3): p. 108-120.
51. Maher, S., et al., *Engineering of micro-to nanostructured 3d-printed drug-releasing titanium implants for enhanced osseointegration and localized delivery of anticancer drugs.* ACS applied materials & interfaces, 2017. **9**(35): p. 29562-29570.
52. Yang, Y., et al., *A 3D-Engineered Conformal Implant Releases DNA Nanocomplexs for Eradicating the Postsurgery Residual Glioblastoma.* Advanced Science, 2017. **4**(8): p. 1600491.
53. Kim, S., et al., *Development of 3D printed applicator in brachytherapy for gynecologic cancer.* International Journal of Radiation Oncology• Biology• Physics, 2017. **99**(2): p. E678.
54. Trout, A.T., et al., *3D printed pathological sectioning boxes to facilitate radiological-pathological correlation in hepatectomy cases.* Journal of Clinical Pathology, 2017. **70**(11): p. 984-987.
55. Wake, N., et al., *3D printed renal cancer models derived from MRI data: application in pre-surgical planning.* Abdominal Radiology, 2017. **42**(5): p. 1501-1509.
56. Samavedi, S. and N. Joy, *3D printing for the development of in vitro cancer models.* Current Opinion in Biomedical Engineering, 2017. **2**: p. 35-42.

57. Barth, R.J., Jr., et al., *A Patient-Specific 3D-Printed Form Accurately Transfers Supine MRI-Derived Tumor Localization Information to Guide Breast-Conserving Surgery.* Annals of Surgical Oncology, 2017. **24**(10): p. 2950-2956.
58. Hamabe, A. and M. Ito, *A three-dimensional pelvic model made with a three-dimensional printer: applications for laparoscopic surgery to treat rectal cancer.* Techniques in Coloproctology, 2017. **21**(5): p. 383-387.
59. Damiati, S., et al., *Acoustic and hybrid 3D-printed electrochemical biosensors for the real-time immunodetection of liver cancer cells (HepG2).* Biosensors and Bioelectronics, 2017. **94**: p. 500-506.
60. Alyaev, Y.G., et al., *Application of 3D printing in urology.* Urologiia (Moscow, Russia : 1999), 2017(4): p. 73-78.
61. Alyaev, Y.G., et al., *Application of 3D soft print models of the kidney for treatment of patients with localized cancer of the kidney (a pilot study).* Urologiia (Moscow, Russia : 1999), 2017(6): p. 12-19.
62. Liu, Y., et al., *Application value of 3D printing technology in the surgery of sphenoid ridge meningioma.* Chinese Journal of Clinical Oncology, 2017. **44**(22): p. 1146-1150.
63. Tang, C.K., A. Vaze, and J.F. Rusling, *Automated 3D-printed unibody immunoarray for chemiluminescence detection of cancer biomarker proteins.* Lab on a Chip, 2017. **17**(3): p. 484-489.
64. Polley, C., et al., *Bioprinting of three dimensional tumor models: A preliminary study using a low cost 3D printer.* Current Directions in Biomedical Engineering, 2017. **3**(2): p. 135-138.
65. Zhao, Y., et al., *Clinical applications of 3-dimensional printing in radiation therapy.* Medical Dosimetry, 2017. **42**(2): p. 150-155.
66. Dai, X., et al., *Coaxial 3D bioprinting of self-assembled multicellular heterogeneous tumor fibers.* Scientific Reports, 2017. **7**(1).
67. Witowski, J.S., et al., *Cost-effective, personalized, 3D-printed liver model for preoperative planning before laparoscopic liver hemihepatectomy for colorectal cancer metastases.* International Journal of Computer Assisted Radiology and Surgery, 2017. **12**(12): p. 2047-2054.
68. Wilke, C.T., et al., *Design and fabrication of a 3D-printed oral stent for head and neck radiotherapy from routine diagnostic imaging.* 3D Print Med, 2017. **3**(1): p. 12.
69. Ji, Z., et al., *Dosimetry verification of radioactive seed implantation for malignant tumors assisted by 3D printing individual templates and CT guidance.* Applied Radiation and Isotopes, 2017. **124**: p. 68-74.
70. Kwakwa, K.A., et al., *Engineering 3D Models of Tumors and Bone to Understand Tumor-Induced Bone Disease and Improve Treatments.* Current Osteoporosis Reports, 2017. **15**(4): p. 247-254.
71. Maher, S., et al., *Engineering of Micro- to Nanostructured 3D-Printed Drug-Releasing Titanium Implants for Enhanced Osseointegration and Localized Delivery of Anticancer Drugs.* ACS Applied Materials and Interfaces, 2017. **9**(35): p. 29562-29570.
72. Kadoya, N., et al., *Evaluation of deformable image registration between external beam radiotherapy and HDR brachytherapy for cervical cancer with a 3D-printed deformable pelvis phantom.* Medical Physics, 2017. **44**(4): p. 1445-1455.
73. Sayed Aluwee, S.A.Z.B., et al., *Evaluation of pre-surgical models for uterine surgery by use of three-dimensional printing and mold casting.* Radiological Physics and Technology, 2017. **10**(3): p. 279-285.
74. Arenas, M., et al., *Individualized 3D scanning and printing for non-melanoma skin cancer brachytherapy: A financial study for its integration into clinical workflow.* Journal of Contemporary Brachytherapy, 2017. **9**(3): p. 270-276.
75. Jones, E.L., et al., *Introduction of novel 3D-printed superficial applicators for high-dose-rate skin brachytherapy.* Brachytherapy, 2017. **16**(2): p. 409-414.
76. Bosc, R., et al., *Mandibular reconstruction after cancer: an in-house approach to manufacturing cutting guides.* International Journal of Oral and Maxillofacial Surgery, 2017. **46**(1): p. 24-31.
77. Li, X., et al., *Multilevel 3D Printing Implant for Reconstructing Cervical Spine with Metastatic Papillary Thyroid Carcinoma.* Spine, 2017. **42**(22): p. E1326-E1330.
78. Wei, R., et al., *One-step reconstruction with a 3D-printed, custom-made prosthesis after total en bloc sacrectomy: a technical note.* European Spine Journal, 2017. **26**(7): p. 1902-1909.
79. Huang, W., et al., *Preliminary application of 3D printing coplanar template in treating pancreatic cancer with 125I seed implantation.* Journal of Interventional Radiology (China), 2017. **26**(11): p. 999-1003.
80. Choy, W.J., et al., *Reconstruction of Thoracic Spine Using a Personalized 3D-Printed Vertebral Body in Adolescent with T9 Primary Bone Tumor.* World Neurosurgery, 2017. **105**: p. 1032.e13-1032.e17.
81. Liang, H., et al., *Reconstruction with 3D-printed pelvic endoprostheses after resection of a pelvic tumour.* Bone and Joint Journal, 2017. **99-B**(2): p. 267-275.
82. Schulz-Wendtland, R., et al., *Semi-automated delineation of breast cancer tumors and subsequent materialization using three-dimensional printing (rapid prototyping).* Journal of Surgical Oncology, 2017. **115**(3): p. 238-242.
83. Choi, Y.R., et al., *Therapeutic response assessment using 3D ultrasound for hepatic metastasis from colorectal cancer: Application of a personalized, 3D-printed tumor model using CT images.* PLoS ONE, 2017. **12**(8).
84. Bartellas, M., et al., *Three-Dimensional Printing of a Hemorrhagic Cervical Cancer Model for Postgraduate Gynecological Training.* Cureus, 2017. **9**(1): p. e950.

85. Andolfi, C., et al., *Usefulness of three-dimensional modeling in surgical planning, resident training, and patient education.* Journal of Laparoendoscopic and Advanced Surgical Techniques, 2017. **27**(5): p. 512-515.
86. George, E., et al., *Utility and reproducibility of 3-dimensional printed models in pre-operative planning of complex thoracic tumors.* Journal of Surgical Oncology, 2017. **116**(3): p. 407-415.
87. Moore, C.A., et al., *3D Bioprinting and Stem Cells.* Methods Mol Biol, 2018. **1842**: p. 93-103.
88. Ahangar, P., et al., *Nanoporous 3D-Printed Scaffolds for Local Doxorubicin Delivery in Bone Metastases Secondary to Prostate Cancer.* Materials, 2018. **11**(9): p. 1485.
89. Ma, H., et al., *3D printing of high-strength bioscaffolds for the synergistic treatment of bone cancer.* NPG Asia Materials, 2018. **10**(4): p. 31-44.
90. Ma, X., et al., *3D bioprinting of functional tissue models for personalized drug screening and in vitro disease modeling.* Advanced Drug Delivery Reviews, 2018. **132**: p. 235-251.
91. Chen, X., et al., *3D printed microfluidic chip for multiple anticancer drug combinations.* Sensors and Actuators, B: Chemical, 2018. **276**: p. 507-516.
92. Jacek, B., et al., *3D printed models in mandibular reconstruction with bony free flaps.* Journal of Materials Science: Materials in Medicine, 2018. **29**(3).
93. Almela, T., et al., *3D printed tissue engineered model for bone invasion of oral cancer.* Tissue and Cell, 2018. **52**: p. 71-77.
94. Yang, N., et al., *3D printing and coating to fabricate a hollow bullet-shaped implant with porous surface for controlled cytoxan release.* International Journal of Pharmaceutics, 2018. **552**(1-2): p. 91-98.
95. Low, L., et al., *3D printing complex lattice structures for permeable liver phantom fabrication.* Bioprinting, 2018. **10**.
96. Afsana, et al., *3D printing in personalized drug delivery.* Current Pharmaceutical Design, 2018. **24**(42): p. 5062-5071.
97. Ozbolat, V., et al., *3D Printing of PDMS Improves Its Mechanical and Cell Adhesion Properties.* ACS Biomaterials Science and Engineering, 2018. **4**(2): p. 682-693.
98. Ma, H., et al., *3D-printed bioceramic scaffolds: From bone tissue engineering to tumor therapy.* Acta Biomaterialia, 2018. **79**: p. 37-59.
99. Liu, Y., et al., *3D-printed scaffolds with bioactive elements-induced photothermal effect for bone tumor therapy.* Acta Biomaterialia, 2018. **73**: p. 531-546.
100. Dang, W., et al., *A bifunctional scaffold with CuFeSe 2 nanocrystals for tumor therapy and bone reconstruction.* Biomaterials, 2018. **160**: p. 92-106.
101. Yang, Y., et al., *An effective thermal therapy against cancer using an E-jet 3D-printing method to prepare implantable magnetocaloric mats.* Journal of Biomedical Materials Research - Part B Applied Biomaterials, 2018. **106**(5): p. 1827-1841.
102. Girolami, M., et al., *Biomimetic 3D-printed custom-made prosthesis for anterior column reconstruction in the thoracolumbar spine: a tailored option following en bloc resection for spinal tumors: Preliminary results on a case-series of 13 patients.* European Spine Journal, 2018. **27**(12): p. 3073-3083.
103. Jiang, T., et al., *Bioprintable alginate/gelatin hydrogel 3D in vitro model systems induce cell spheroid formation.* Journal of Visualized Experiments, 2018. **2018**(137).
104. Wang, X., et al., *Bioprinting of glioma stem cells improves their endotheliogenic potential.* Colloids and Surfaces B: Biointerfaces, 2018. **171**: p. 629-637.
105. Wang, B., et al., *Computer-aided designed, three dimensional-printed hemipelvic prosthesis for peri-acetabular malignant bone tumour.* International Orthopaedics, 2018. **42**(3): p. 687-694.
106. Dupret-Bories, A., et al., *Contribution of 3D printing to mandibular reconstruction after cancer.* European Annals of Otorhinolaryngology, Head and Neck Diseases, 2018. **135**(2): p. 133-136.
107. Porpiglia, F., et al., *Current Use of Three-dimensional Model Technology in Urology: A Road Map for Personalised Surgical Planning.* European Urology Focus, 2018. **4**(5): p. 652-656.
108. Ferreira, L.P., V.M. Gaspar, and J.F. Mano, *Design of spherically structured 3D in vitro tumor models -Advances and prospects.* Acta Biomaterialia, 2018. **75**: p. 11-34.
109. Chen, S., et al., *Electrospinning: An enabling nanotechnology platform for drug delivery and regenerative medicine.* Advanced Drug Delivery Reviews, 2018. **132**: p. 188-213.
110. Wong, J.K., et al., *Emerging In Vitro 3D Tumour Models in Nanoparticle-Based Gene and Drug Therapy.* Trends in Biotechnology, 2018. **36**(5): p. 477-480.
111. Rodrigues, T., et al., *Emerging tumor spheroids technologies for 3D in vitro cancer modeling.* Pharmacology and Therapeutics, 2018. **184**: p. 201-211.
112. Qiao, H. and T. Tang, *Engineering 3D approaches to model the dynamic microenvironments of cancer bone metastasis.* Bone Research, 2018. **6**(1).
113. Hoarau-Véchot, J., et al., *Halfway between 2D and animal models: Are 3D cultures the ideal tool to study cancer-microenvironment interactions?* International Journal of Molecular Sciences, 2018. **19**(1).
114. Dzian, A., et al., *Implantation of a 3D-printed titanium sternum in a patient with a sternal tumor.* World Journal of Surgical Oncology, 2018. **16**(1).

115. Zhong, J., et al., *In Vitro Study of Colon Cancer Cell Migration Using E-Jet 3D Printed Cell Culture Platforms.* Macromolecular Bioscience, 2018. **18**(11).
116. Pravin, S. and A. Sudhir, *Integration of 3D printing with dosage forms: A new perspective for modern healthcare.* Biomedicine and Pharmacotherapy, 2018. **107**: p. 146-154.
117. van Pel, D.M., et al., *Modelling glioma invasion using 3D bioprinting and scaffold-free 3D culture.* Journal of Cell Communication and Signaling, 2018. **12**(4): p. 723-730.
118. Chiu, T.D., et al., *MR-CBCT image-guided system for radiotherapy of orthotopic rat prostate tumors.* PLoS ONE, 2018. **13**(5).
119. van de Belt, T.H., et al., *Patient-Specific Actual-Size Three-Dimensional Printed Models for Patient Education in Glioma Treatment: First Experiences.* World Neurosurg, 2018. **117**: p. e99-e105.
120. Weinberg, L., et al., *Personalised 3D-printed model of a chest-wall chondrosarcoma to enhance patient understanding of complex cardiothoracic surgery.* BMJ Case Reports, 2018. **2018**.
121. Rao, N., et al., *Proof-of-Concept Study of 3-D–Printed Mold–Guided Breast-Conserving Surgery in Breast Cancer Patients.* Clinical Breast Cancer, 2018. **18**(5): p. e769-e772.
122. Ma, X., et al., *Rapid 3D bioprinting of decellularized extracellular matrix with regionally varied mechanical properties and biomimetic microarchitecture.* Biomaterials, 2018. **185**: p. 310-321.
123. Polonio-Alcalá, E., et al., *Screening of additive manufactured scaffolds designs for triple negative breast cancer 3D cell culture and stem-like expansion.* International Journal of Molecular Sciences, 2018. **19**(10).
124. Liang, Y., et al., *Three-dimensional-printed individual template-guided 125I seed implantation for the cervical lymph node metastasis: A dosimetric and security study.* Journal of Cancer Research and Therapeutics, 2018. **14**(1): p. 30-35.
125. Wang, X., et al., *Tumor-like lung cancer model based on 3D bioprinting.* 3 Biotech, 2018. **8**(12).
126. Hazelaar, C., et al., *Using 3D printing techniques to create an anthropomorphic thorax phantom for medical imaging purposes.* Medical Physics, 2018. **45**(1): p. 92-100.
127. Lin, J., et al., *Using Three-Dimensional Printing to Create Individualized Cranial Nerve Models for Skull Base Tumor Surgery.* World Neurosurgery, 2018. **120**: p. e142-e152.
128. Yoon, S.H., et al., *Virtual reality-assisted localization and three-dimensional printing-enhanced multidisciplinary decision to treat radiologically occult superficial endobronchial lung cancer.* Thoracic Cancer, 2018. **9**(11): p. 1525-1527.
129. Ehler, E., et al., *Workload implications for clinic workflow with implementation of three-dimensional printed customized bolus for radiation therapy: A pilot study.* PLoS ONE, 2018. **13**(10).
130. Wang, X., et al., *3D bioprinted glioma cell-laden scaffolds enriching glioma stem cells via epithelial–mesenchymal transition.* Journal of Biomedical Materials Research - Part A, 2019. **107**(2): p. 383-391.
131. Qiao, X., et al., *E-Jet 3D-Printed Scaffolds as Sustained Multi-Drug Delivery Vehicles in Breast Cancer Therapy.* Pharmaceutical research, 2019. **36**(12): p. 182.
132. Shi, K., et al., *Drop-on-powder 3d printing of tablets with an anti-cancer drug, 5-fluorouracil.* Pharmaceutics, 2019. **11**(4): p. 150.
133. Chmura, J., et al., *Novel design and development of a 3D-printed conformal superficial brachytherapy device for the treatment of non-melanoma skin cancer and keloids.* 3D printing in medicine, 2019. **5**(1): p. 10.
134. Hosseinzadeh, R., et al., *A Drug-Eluting 3D-Printed Mesh (GlioMesh) for Management of Glioblastoma.* Advanced Therapeutics, 2019.
135. Meng, F., et al., *3D Bioprinted In Vitro Metastatic Models via Reconstruction of Tumor Microenvironments.* Advanced Materials, 2019. **31**(10).
136. Zahedi-Tabar, Z., et al., *3D in vitro cancerous tumor models: Using 3D printers.* Medical Hypotheses, 2019. **124**: p. 91-94.
137. Luo, Y., et al., *3D printing of hydrogel scaffolds for future application in photothermal therapy of breast cancer and tissue repair.* Acta Biomaterialia, 2019. **92**: p. 37-47.
138. Cho, H., et al., *3D Printing of Poloxamer 407 Nanogel Discs and Their Applications in Adjuvant Ovarian Cancer Therapy.* Molecular Pharmaceutics, 2019. **16**(2): p. 552-560.
139. Heinrich, M.A., et al., *3D-Bioprinted Mini-Brain: A Glioblastoma Model to Study Cellular Interactions and Therapeutics.* Advanced Materials, 2019. **31**(14).
140. He, Y., et al., *3D-printed breast phantom for multi-purpose and multi-modality imaging.* Quantitative Imaging in Medicine and Surgery, 2019. **9**(1): p. 63-74.
141. Feng, D., et al., *3D-printed prosthesis replacement for limb salvage after radical resection of an ameloblastoma in the tibia with 1 year of follow up: A case report.* Yonsei Medical Journal, 2019. **60**(9): p. 882-886.
142. Mirani, B., et al., *A 3D bioprinted hydrogel mesh loaded with all-trans retinoic acid for treatment of glioblastoma.* European Journal of Pharmacology, 2019. **854**: p. 201-212.
143. Zanoni, M., et al., *Anticancer drug discovery using multicellular tumor spheroid models.* Expert Opinion on Drug Discovery, 2019. **14**(3): p. 289-301.

144. Saglam-Metiner, P., S. Gulce-Iz, and C. Biray-Avci, *Bioengineering-inspired three-dimensional culture systems: Organoids to create tumor microenvironment.* Gene, 2019. **686**: p. 203-212.
145. Lee, C., et al., *Bioprinting a novel glioblastoma tumor model using a fibrin-based bioink for drug screening.* Materials Today Chemistry, 2019. **12**: p. 78-84.
146. Liu, X., et al., *Combined Application of Modified Three-Dimensional Printed Anatomic Templates and Customized Cutting Blocks in Pelvic Reconstruction After Pelvic Tumor Resection.* Journal of Arthroplasty, 2019. **34**(2): p. 338-345.e1.
147. Zaid, M., et al., *Creating customized oral stents for head and neck radiotherapy using 3D scanning and printing.* Radiation Oncology, 2019. **14**(1).
148. Roberts, S., S. Peyman, and V. Speirs, *Current and Emerging 3D Models to Study Breast Cancer.* Adv Exp Med Biol, 2019. **1152**: p. 413-427.
149. Witowski, J., et al., *Decision-making based on 3D printed models in laparoscopic liver resections with intraoperative ultrasound: a prospective observational study.* Eur Radiol, 2019.
150. Logar, H.B.Z., R. Hudej, and B. Šegedin, *Development and assessment of 3D-printed individual applicators in gynecological MRI-guided brachytherapy.* Journal of Contemporary Brachytherapy, 2019. **11**(2): p. 128-136.
151. Lecornu, M., et al., *Digital applicator by 3D printing in contact brachytherapy.* Cancer/Radiotherapie, 2019. **23**(4): p. 328-333.
152. Kitamori, H., et al., *Evaluation of mouthpiece fixation devices for head and neck radiotherapy patients fabricated in PolyJet photopolymer by a 3D printer.* Physica Medica, 2019. **58**: p. 90-98.
153. Campos, D.F.D., et al., *Exploring cancer cell behavior in vitro in three-dimensional multicellular bioprintable collagen-based hydrogels.* Cancers, 2019. **11**(2).
154. Cacciamani, G.E., et al., *Impact of Three-dimensional Printing in Urology: State of the Art and Future Perspectives. A Systematic Review by ESUT-YAUWP Group.* European Urology, 2019. **76**(2): p. 209-221.
155. Aristei, C., et al., *Individualized 3D-printed templates for high-dose-rate interstitial multicathether brachytherapy in patients with breast cancer.* Brachytherapy, 2019. **18**(1): p. 57-62.
156. Kingsley, D.M., et al., *Laser-based 3D bioprinting for spatial and size control of tumor spheroids and embryoid bodies.* Acta Biomaterialia, 2019. **95**: p. 357-370.
157. Langer, E.M., et al., *Modeling Tumor Phenotypes In Vitro with Three-Dimensional Bioprinting.* Cell Reports, 2019. **26**(3): p. 608-623.e6.
158. Yoon, S.H., et al., *Personalized 3D-Printed Model for Informed Consent for Stage I Lung Cancer: A Randomized Pilot Trial.* Seminars in Thoracic and Cardiovascular Surgery, 2019. **31**(2): p. 316-318.
159. Ji, Z., et al., *Safety and efficacy of CT-guided radioactive iodine-125 seed implantation assisted by a 3D printing template for the treatment of thoracic malignancies.* Journal of Cancer Research and Clinical Oncology, 2019.
160. Emile, S.H. and S.D. Wexner, *Systematic review of the applications of three-dimensional printing in colorectal surgery.* Colorectal Disease, 2019. **21**(3): p. 261-269.
161. Santiago, L., et al., *The role of three-dimensional printing in the surgical management of breast cancer.* Journal of Surgical Oncology, 2019. **120**(6): p. 897-902.
162. Hermida, M.A., et al., *Three dimensional in vitro models of cancer: Bioprinting multilineage glioblastoma models*, in *Advances in Biological Regulation*. 2019, Elsevier Ltd.
163. Fan, G., et al., *Three-dimensional printing for laparoscopic partial nephrectomy in patients with renal tumors.* Journal of International Medical Research, 2019. **47**(9): p. 4324-4332.
164. Lee, J.H., et al., *Three-dimensional-printed vaginal applicators for electronic brachytherapy of endometrial cancers.* Medical Physics, 2019. **46**(2): p. 448-455.
165. Angelini, A., et al., *Three-dimension-printed custom-made prosthetic reconstructions: from revision surgery to oncologic reconstructions.* International Orthopaedics, 2019. **43**(1): p. 123-132.
166. Schmidt, S.K., et al., *Tumor Cells Develop Defined Cellular Phenotypes After 3D-Bioprinting in Different Bioinks.* Cells, 2019. **8**(10).
167. Han, S., et al., *3D bioprinted vascularized tumour for drug testing.* International Journal of Molecular Sciences, 2020. **21**(8).
168. Shee, K., et al., *A novel ex vivo trainer for robotic vesicourethral anastomosis.* Journal of Robotic Surgery, 2020. **14**(1): p. 21-27.
169. Kang, Y., et al., *3D Bioprinting of Tumor Models for Cancer Research.* ACS Applied Bio Materials, 2020. **3**(9): p. 5552-5573.
170. Wang, Y., et al., *3D printed biodegradable implants as an individualized drug delivery system for local chemotherapy of osteosarcoma.* Materials and Design, 2020. **186**.
171. Wei, X., et al., *3D printed core-shell hydrogel fiber scaffolds with NIR-triggered drug release for localized therapy of breast cancer.* International Journal of Pharmaceutics, 2020. **580**.
172. Chen, H., et al., *3D printed in vitro tumor tissue model of colorectal cancer.* Theranostics, 2020. **10**(26): p. 12127-12143.

173. Chen, J., et al., *3D printed microfluidic devices for circulating tumor cells (CTCs) isolation.* Biosensors and Bioelectronics, 2020. **150**.
174. Uddin, M.J., et al., *3D printed microneedles for anticancer therapy of skin tumours.* Materials Science and Engineering C, 2020. **107**.
175. Haleem, A., M. Javaid, and R. Vaishya, *3D printing applications for the treatment of cancer.* Clinical Epidemiology and Global Health, 2020.
176. Dang, W., et al., *3D printing of metal-organic framework nanosheets-structured scaffolds with tumor therapy and bone construction.* Biofabrication, 2020. **12**(2).
177. Wake, N., et al., *3D Printing, Augmented Reality, and Virtual Reality for the Assessment and Management of Kidney and Prostate Cancer: A Systematic Review.* Urology, 2020.
178. Sharafeldin, M., et al., *3D-printed immunosensor arrays for cancer diagnostics.* Sensors (Switzerland), 2020. **20**(16): p. 1-23.
179. Zaid, M., et al., *A prospective parallel design study testing non-inferiority of customized oral stents made using 3D printing or manually fabricated methods.* Oral Oncology, 2020. **106**.
180. Asfia, A., et al., *A review of 3D printed patient specific immobilisation devices in radiotherapy.* Physics and Imaging in Radiation Oncology, 2020. **13**: p. 30-35.
181. Moghaddam, S.H.Z., et al., *A review on the application of 3d printing technology in radiotherapy for breast cancer.* Journal of Mazandaran University of Medical Sciences, 2020. **30**(188): p. 185-200.
182. Abasalizadeh, F., et al., *Alginate-based hydrogels as drug delivery vehicles in cancer treatment and their applications in wound dressing and 3D bioprinting.* J Biol Eng, 2020. **14**: p. 8.
183. Angelini, A., et al., *Analysis of principles inspiring design of three-dimensional-printed custom-made prostheses in two referral centres.* International Orthopaedics, 2020. **44**(5): p. 829-837.
184. Lan, Q., et al., *Application of 3D-Printed Craniocerebral Model in Simulated Surgery for Complex Intracranial Lesions.* World Neurosurgery, 2020. **134**: p. e761-e770.
185. Sun, L., et al., *Application of a 3D Bioprinted Hepatocellular Carcinoma Cell Model in Antitumor Drug Research.* Frontiers in Oncology, 2020. **10**.
186. Soman, S.S. and S. Vijayavenkataraman, *Applications of 3D bioprinted-induced pluripotent stem cells in healthcare.* International Journal of Bioprinting, 2020. **6**(4).
187. Chaji, S., J. Al-Saleh, and C.T. Gomillion, *Bioprinted Three-Dimensional Cell-Laden Hydrogels to Evaluate Adipocyte-Breast Cancer Cell Interactions.* Gels, 2020. **6**(1).
188. Mao, S., et al., *Bioprinting of <i>in vitro</i> tumor models for personalized cancer treatment: a review.* Biofabrication, 2020.
189. Mao, S., et al., *Bioprinting of patient-derivedin vitrointrahepatic cholangiocarcinoma tumor model: establishment, evaluation and anti-cancer drug testing.* Biofabrication, 2020.
190. Bahcecioglu, G., et al., *Breast cancer models: Engineering the tumor microenvironment.* Acta Biomaterialia, 2020. **106**: p. 1-21.
191. Wang, F.M., et al., *Clinical observation of 3D printing-guided three-dimensional brachytherapy for cervical cancer.* Chinese Journal of Cancer Prevention and Treatment, 2020. **27**(12): p. 1003-1007.
192. Brancato, V., et al., *Could 3D models of cancer enhance drug screening?* Biomaterials, 2020. **232**: p. 119744.
193. Wang, C., et al., *Cryogenic 3D printing of porous scaffolds for in situ delivery of 2D black phosphorus nanosheets, doxorubicin hydrochloride and osteogenic peptide for treating tumor resection-induced bone defects.* Biofabrication, 2020. **12**(3).
194. Mussi, E., et al., *Current practice in preoperative virtual and physical simulation in neurosurgery.* Bioengineering, 2020. **7**(1).
195. Witowski, J., et al., *Decision-making based on 3D printed models in laparoscopic liver resections with intraoperative ultrasound: a prospective observational study.* European Radiology, 2020. **30**(3): p. 1306-1312.
196. Shafiee, A., *Design and Fabrication of Three-Dimensional Printed Scaffolds for Cancer Precision Medicine.* Tissue Engineering - Part A, 2020. **26**(5-6): p. 305-317.
197. Zhao, C., et al., *Design, modeling and 3D printing of a personalized cervix tissue implant with protein release function.* Biomedical materials (Bristol, England), 2020. **15**(4): p. 045005.
198. Li, N.T., et al., *Development of a bioprinting approach for automated manufacturing of multi-cell type biocomposite TRACER strips using contact capillary-wicking.* Biofabrication, 2020. **12**(1).
199. Yang, Y., et al., *E-jet 3D printed drug delivery implants to inhibit growth and metastasis of orthotopic breast cancer.* Biomaterials, 2020. **230**.
200. Cui, H., et al., *Engineering a Novel 3D Printed Vascularized Tissue Model for Investigating Breast Cancer Metastasis to Bone.* Advanced Healthcare Materials, 2020. **9**(15).
201. Jiang, T., et al., *Engineering bioprintable alginate/gelatin composite hydrogels with tunable mechanical and cell adhesive properties to modulate tumor spheroid growth kinetics.* Biofabrication, 2020. **12**(1).
202. Ruiz-Garcia, H., et al., *Engineering Three-Dimensional Tumor Models to Study Glioma Cancer Stem Cells and Tumor Microenvironment.* Frontiers in Cellular Neuroscience, 2020. **14**.

203. Molley, T.G., et al., *Geometrically Structured Microtumors in 3D Hydrogel Matrices.* Adv Biosyst, 2020. **4**(5): p. e2000056.
204. Maloney, E., et al., *Immersion bioprinting of tumor organoids in multi-well plates for increasing chemotherapy screening throughput.* Micromachines, 2020. **11**(2).
205. Jovičić, M.Š., et al., *Implementation of the three-dimensional printing technology in treatment of bone tumours: a case series.* International Orthopaedics, 2021. **45**(4): p. 1079-1085.
206. Li, J., et al., *Improving Bioprinted Volumetric Tumor Microenvironments In Vitro.* Trends in Cancer, 2020. **6**(9): p. 745-756.
207. Liu, T., et al., *Investigating lymphangiogenesis in a sacrificially bioprinted volumetric model of breast tumor tissue.* Methods, 2020.
208. Hakobyan, D., et al., *Laser-assisted 3D bioprinting of exocrine pancreas spheroid models for cancer initiation study.* Biofabrication, 2020. **12**(3).
209. Montejo Maillo, B., et al. *Methodology of custom design and manufacturing of 3D external breast prostheses*. in *ACM International Conference Proceeding Series*. 2020.
210. Chen, M.Y., et al., *Multi-colour extrusion fused deposition modelling: a low-cost 3D printing method for anatomical prostate cancer models.* Scientific Reports, 2020. **10**(1).
211. Campelo, S., et al., *Multimaterial three-dimensional printing in brachytherapy: Prototyping teaching tools for interstitial and intracavitary procedures in cervical cancers.* Brachytherapy, 2020. **19**(6): p. 767-776.
212. Choe, G., R. Carr, and D. Molena, *New Surgical Approaches in the Treatment of Non–Small Cell Lung Cancer.* Clinics in Chest Medicine, 2020. **41**(2): p. 175-183.
213. Shi, K., et al., *Novel 3D printed device with integrated macroscale magnetic field triggerable anti-cancer drug delivery system.* Colloids and Surfaces B: Biointerfaces, 2020. **192**.
214. Radhakrishnan, J., et al., *Organotypic cancer tissue models for drug screening: 3D constructs, bioprinting and microfluidic chips.* Drug Discovery Today, 2020. **25**(5): p. 879-890.
215. Ramzy, G.M., et al., *Patient-Derived In Vitro Models for Drug Discovery in Colorectal Carcinoma.* Cancers (Basel), 2020. **12**(6).
216. Bae, J., S. Han, and S. Park, *Recent Advances in 3D Bioprinted Tumor Microenvironment.* Biochip Journal, 2020. **14**(2): p. 137-147.
217. Oztan, Y.C., et al., *Recent Advances on Utilization of Bioprinting for Tumor Modeling.* Bioprinting, 2020. **18**.
218. Ma, L., et al., *The construction of in vitro tumor models based on 3D bioprinting.* Bio-Design and Manufacturing, 2020.
219. Wang, Y., et al., *The role of a drug-loaded poly (lactic co-glycolic acid) (PLGA) copolymer stent in the treatment of ovarian cancer.* Cancer Biology and Medicine, 2020. **17**(1): p. 237-250.
220. Dang, H.P., D.W. Hutmacher, and P.A. Tran. *The Use of 3D Printed Microporous-Strut Polycaprolactone Scaffolds for Targeted Local Delivery of Chemotherapeutic Agent for Breast Cancer Application*. 2020. Springer Verlag.
221. Campbell, A., et al., *Thermal Bioprinting Causes Ample Alterations of Expression of LUCAT1, IL6, CCL26, and NRN1L Genes and Massive Phosphorylation of Critical Oncogenic Drug Resistance Pathways in Breast Cancer Cells.* Frontiers in Bioengineering and Biotechnology, 2020. **8**.
222. Hermida, M.A., et al., *Three dimensional in vitro models of cancer: Bioprinting multilineage glioblastoma models.* Adv Biol Regul, 2020. **75**: p. 100658.
223. Tang, M., et al., *Three-dimensional bioprinted glioblastoma microenvironments model cellular dependencies and immune interactions.* Cell Research, 2020.
224. Huang, Y.M., et al., *Three-dimensional printed silicone bite blocks for radiotherapy of head and neck cancer-a preliminary study.* Applied Sciences (Switzerland), 2020. **10**(5).
225. Smelt, J., et al., *Three-Dimensional Printing for Chest Wall Reconstruction in Thoracic Surgery: Building on Experience.* Thoracic and Cardiovascular Surgeon, 2020. **68**(4): p. 352-356.
226. Qiu, B., et al., *Three-dimensional reconstruction/personalized three-dimensional printed model for thoracoscopic anatomical partial-lobectomy in stage i lung cancer: A retrospective study.* Translational Lung Cancer Research, 2020. **9**(4): p. 1235-1246.
227. Lador, R., et al., *Use of 3-Dimensional Printing Technology in Complex Spine Surgeries.* World Neurosurgery, 2020. **133**: p. e327-e341.
228. Michiels, C., et al., *Use of personalized 3D printed kidney models for partial nephrectomy.* Progres en Urologie, 2020. **30**(15): p. 970-975.
229. Hong, D., et al., *Usefulness of a 3D-Printed Thyroid Cancer Phantom for Clinician to Patient Communication.* World Journal of Surgery, 2020. **44**(3): p. 788-794.
230. Augustine, R., et al., *3D Bioprinted cancer models: Revolutionizing personalized cancer therapy.* Translational Oncology, 2021. **14**(4).
231. Almela, T., L. Tayebi, and K. Moharamzadeh, *3D bioprinting for in vitro models of oral cancer: Toward development and validation.* Bioprinting, 2021. **22**.

232. Sharifi, M., et al., *3D bioprinting of engineered breast cancer constructs for personalized and targeted cancer therapy.* Journal of Controlled Release, 2021. **333**: p. 91-106.
233. Bojin, F., et al., *3D Bioprinting of Model Tissues That Mimic the Tumor Microenvironment.* Micromachines (Basel), 2021. **12**(5).
234. Mu, X., J. Zhang, and Y. Jiang, *3D Printing in Breast Reconstruction: From Bench to Bed.* Frontiers in Surgery, 2021. **8**.
235. Herreros-Pomares, A., et al., *3D printing novel in vitro cancer cell culture model systems for lung cancer stem cell study.* Materials Science and Engineering C, 2021. **122**.
236. Moore, C.A., et al., *A 3D Bioprinted Material That Recapitulates the Perivascular Bone Marrow Structure for Sustained Hematopoietic and Cancer Models.* Polymers (Basel), 2021. **13**(4).
237. Santiago, L., et al., *Acceptability of 3D-printed breast models and their impact on the decisional conflict of breast cancer patients: A feasibility study.* J Surg Oncol, 2021. **123**(5): p. 1206-1214.
238. Orcheston-Findlay, L., et al., *Advanced Spheroid, Tumouroid and 3D Bioprinted In-Vitro Models of Adult and Paediatric Glioblastoma.* Int J Mol Sci, 2021. **22**(6).
239. Sánchez-Salazar, M.G., M.M. Álvarez, and G. Trujillo-de Santiago, *Advances in 3D bioprinting for the biofabrication of tumor models.* Bioprinting, 2021. **21**.
240. Flores-Torres, S., et al., *Alginate-gelatin-Matrigel hydrogels enable the development and multigenerational passaging of patient-derived 3D bioprinted cancer spheroid models.* Biofabrication, 2021. **13**(2).
241. Quinn, C.H., A.M. Beierle, and E.A. Beierle, *Artificial Tumor Microenvironments in Neuroblastoma.* Cancers (Basel), 2021. **13**(7).
242. Horder, H., et al., *Bioprinting and Differentiation of Adipose-Derived Stromal Cell Spheroids for a 3D Breast Cancer-Adipose Tissue Model.* Cells, 2021. **10**(4).
243. Suarez-Martinez, A.D., et al., *Bioprinting on Live Tissue for Investigating Cancer Cell Dynamics.* Tissue Engineering - Part A, 2021. **27**(7-8): p. 438-453.
244. Lobo, D.A., et al., *Cancer Cell Direct Bioprinting: A Focused Review.* Micromachines (Basel), 2021. **12**(7).
245. Michiels, C., et al., *Comprehensive review of 3D printing use in medicine: Comparison with practical applications in urology.* Progres en Urologie, 2021.
246. Miranda, M.A., et al., *Cytotoxic and chemosensitizing effects of glycoalkaloidic extract on 2D and 3D models using RT4 and patient derived xenografts bladder cancer cells.* Mater Sci Eng C Mater Biol Appl, 2021. **119**: p. 111460.
247. Sharafeldin, M., et al., *Detecting cancer metastasis and accompanying protein biomarkers at single cell levels using a 3D-printed microfluidic immunoarray.* Biosens Bioelectron, 2021. **171**: p. 112681.
248. Feng, G., et al., *Development of A 3D-Printed Navigational Template for Establishing Rabbit VX2 Lung Cancer Model.* J Surg Res, 2021. **267**: p. 358-365.
249. Ju, S.G., et al., *Development of a Tongue Immobilization Device Using a 3D Printer for Intensity Modulated Radiation Therapy of Nasopharyngeal Cancer Patients.* Cancer Res Treat, 2021. **53**(1): p. 45-54.
250. Ormandy, D., et al., *Difficult airways: a 3D printing study with virtual fibreoptic endoscopy.* British Journal of Oral and Maxillofacial Surgery, 2021. **59**(2): p. e65-e71.
251. Wang, L., et al., *Dosimetric comparison of computed tomography-guided iodine-125 seed implantation assisted with and without three-dimensional printing non-coplanar template in locally recurrent rectal cancer: a propensity score matching study.* J Contemp Brachytherapy, 2021. **13**(1): p. 18-23.
252. Ruiz-Espigares, J., et al., *Evolution of Metastasis Study Models toward Metastasis-On-A-Chip: The Ultimate Model?* Small, 2021. **17**(14).
253. Yi, H.G., *Introduction to bioprinting of in vitro cancer models.* Essays Biochem, 2021.
254. Morales, X., I. Cortés-Domínguez, and C. Ortiz-De-Solorzano, *Modeling the mechanobiology of cancer cell migration using 3D biomimetic hydrogels.* Gels, 2021. **7**(1): p. 1-35.
255. Ghazi, A., et al., *Multi-institutional validation of a perfused robot-assisted partial nephrectomy procedural simulation platform utilizing clinically relevant objective metrics of simulators (CROMS).* BJU International, 2021. **127**(6): p. 645-653.
256. Garcia-Sevilla, M., et al., *Patient-specific desktop 3D-printed guides for pelvic tumour resection surgery: a precision study on cadavers.* Int J Comput Assist Radiol Surg, 2021. **16**(3): p. 397-406.
257. Choi, Y., et al., *Patient-Specific Quality Assurance Using a 3D-Printed Chest Phantom for Intraoperative Radiotherapy in Breast Cancer.* Front Oncol, 2021. **11**: p. 629927.
258. Hughes, A.M., et al., *Printing the Pathway Forward in Bone Metastatic Cancer Research: Applications of 3D Engineered Models and Bioprinted Scaffolds to Recapitulate the Bone-Tumor Niche.* Cancers (Basel), 2021. **13**(3).
259. Tang, J., et al., *Programmable shape transformation of 3D printed magnetic hydrogel composite for hyperthermia cancer therapy.* Extreme Mechanics Letters, 2021. **46**.
260. Andersen, B.T., et al., *Re-interpreting mesenteric vascular anatomy on 3D virtual and/or physical models: positioning the middle colic artery bifurcation and its relevance to surgeons operating colon cancer.* Surg Endosc, 2021.
261. Dey, M., et al., *Studying Tumor Angiogenesis and Cancer Invasion in a Three-Dimensional Vascularized Breast Cancer Micro-Environment.* Advanced Biology, 2021.

262. Jiang, W., et al., *The accuracy and dosimetric analysis of 3D-printing non-coplanar template-assisted iodine-125 seed implantation for recurrent chest wall cancer.* Journal of Contemporary Brachytherapy, 2021. **13**(3): p. 273-279.
263. Qiu, B., et al., *The Accuracy of Individualized 3D-Printing Template-Assisted I125 Radioactive Seed Implantation for Recurrent/Metastatic Head and Neck Cancer.* Frontiers in Oncology, 2021. **11**.
264. Wang, H., et al., *The dosimetry evaluation of 3D printing non-coplanar template-assisted CT-guided 125I seed stereotactic ablation brachytherapy for pelvic recurrent rectal cancer after external beam radiotherapy.* Journal of radiation research, 2021. **62**(3): p. 473-482.
265. Hu, W., et al., *Three-dimensional computed tomography angiography and bronchography combined with three-dimensional printing for thoracoscopic pulmonary segmentectomy in stage IA non-small cell lung cancer.* Journal of Thoracic Disease, 2021. **13**(2): p. 1187-1195.
266. Gan, Y., et al., *Total Talar Replacement Using a Novel 3D-Printed Prosthesis for Recurrent Giant Cell Tumour of the Talus.* Journal of Shanghai Jiaotong University (Science), 2021. **26**(3): p. 391-397.
